# G65V Substitution in Actin Disturbs Polymerization Leading to Inhibited Cell Elongation in Cotton

**DOI:** 10.3389/fpls.2019.01486

**Published:** 2019-11-15

**Authors:** Yongwang Sun, Wenhua Liang, Weijuan Shen, Hao Feng, Jiedan Chen, Zhanfeng Si, Yan Hu, Tianzhen Zhang

**Affiliations:** ^1^State Key Laboratory of Crop Genetics and Germplasm Enhancement, Cotton Hybrid R & D Engineering Center (the Ministry of Education), College of Agriculture, Nanjing Agricultural University, Nanjing, China; ^2^Zhejiang Provincial Key Laboratory of Crop Genetic Resources, Institute of Crop Science, Plant Precision Breeding Academy, College of Agriculture and Biotechnology, Zhejiang University, Zhejiang, China

**Keywords:** actin polymerization, cell elongation, cotton, map-based cloning, plant morphology, short fiber

## Abstract

The importance of the actin cytoskeleton for proper cell development has been well established in a variety of organisms. Actin protein sequences are highly conserved, and each amino acid residue may be essential for its function. In this study, we report the isolation and characterization of *GhLi* *_1_* from an upland cotton mutant Ligon lintless-1 (Li_1_), which harbors the G65V substitution in its encoded actin protein. Li_1_ mutants exhibit pleiotropic malformed phenotypes, including dwarf plants, distorted organs, and extremely shortened fibers. Cytological analysis showed that the actin cytoskeleton was disorganized and the abundance of F-actin was decreased in the Li_1_ cells. Vesicles were aggregated into patches, and excessive cellulose synthase complexes were inserted into the plasma membrane during the secondary cell wall biosynthesis stage, which dramatically affected the morphology of the Li_1_ cells. Molecular model prediction suggested that the G65V substitution may affect the three-bodied G-actin interaction during F-actin assembly. Biochemical assays demonstrated that the recombinant GhLi_1_ protein disturbs actin dynamics by inhibiting the nucleation and elongation processes. Therefore, our findings demonstrate that the G65V substitution in actin had dominant-negative effects on cell elongation, by disturbing actin polymerization and actin cytoskeleton-based biological processes such as intracellular transportation.

## Introduction

The actin cytoskeleton is a fundamental and dynamic network in eukaryotic cells. It is not only involved in the maintenance of cell shape and structure, but also regulates a tremendous range of cellular processes, including cytoplasmic streaming, organelle movement, cell expansion, cell wall deposition and responses to internal and external signals (Staiger et al., 2000; Hussey et al., 2006). Actin exists in cells in a dynamic equilibrium between two principal forms: globular monomeric actin (G-actin) and filamentous polymeric actin (F-actin). Most of actin’s biological functions are conducted by F-actin, which is assembled from G-actin subunits into a helical structure polymer (Holmes et al., 1990). A sophisticated regulatory system, represented by a plethora of actin binding proteins (ABPs), has developed to modulate actin dynamics, including assembly and disassembly of F-actins, and also their organization into higher-order networks (Blanchoin et al., 2010). The highly dynamic and widely distributed actin cytoskeleton allows prompt responses of the cell to signals arising from developmental and environmental stimuli, ensuring the precise regulation of cell development (Staiger et al., 2000; Blanchoin et al., 2010).

Actin protein sequences vary little in length from 377 amino acid residues and share more than 80% protein sequence similarity (Šlajcherová et al., 2012). Amino acid substitutions in actin, especially those that have an impact on their surface properties, are very likely to alter actin-actin or actin-ABP interactions (McDowell et al., 1996; Feng and Marston, 2009). Numerous actin mutants caused by amino acid substitution have been found in both uni- and multi-cellular organisms. Some yeast actin mutations result in growth deficiencies, or even death of the cell (Ishiguro and Kobayashi, 1996; Teal and Dawson, 2007). In humans, many actin mutations have been found to be pathogenetic, and result in a range of congenital disorders, such as myopathy (Feng and Marston, 2009; Marston, 2018) and deafness (Bryan et al., 2006; Morin et al., 2009), depending on the site of mutation and the type of actin affected. In Arabidopsis, amino acid substitution in actin led to disruption of the F-actin network, resulting in various morphological malformations, such as dwarf plants and deformed organs (Ringli et al., 2002; Nishimura et al., 2003; Kato et al., 2010).

During plant cell growth, cell wall matrix precursors and membrane materials are constructed in the Golgi system, and then delivered to the existing cell wall at the site of expansion *via* exocytotic vesicles (Emons and Ketelaar, 2009). The membranes of these vesicles fuse with the plasma membrane (PM) causing the insertion of transmembrane proteins, typically represented by the cellulose microfibril-producing cellulose synthase complex (CSC), into the PM (Gardiner et al., 2003; Emons and Ketelaar, 2009). The importance of the actin cytoskeleton in plant cell growth had been well established by numerous pharmacological and genetic studies (Szymanski et al., 1999; Chen et al., 2009; Pei et al., 2012), and a growing body of evidence has confirmed that the actin cytoskeleton is essential in supporting the intracellular movement of cytoplasmic organelles (Miller et al., 1999; Mathur and Martin, 2002; Hussey et al., 2006; Akkerman et al., 2012). Any perturbation of the actin cytoskeleton by application of actin-depolymerizing drugs (Miller et al., 1999; Szymanski et al., 1999; Ketelaar et al., 2003; Chen et al., 2009), or spontaneous mutation or genetic manipulation of actin cytoskeleton related genes (Chen et al., 2002; Mathur and Martin, 2002; Nishimura et al., 2003; Wang et al., 2009; Kato et al., 2010; Yang et al., 2011; Zhang et al., 2011; Wu et al., 2015) affects cell elongation, expansion, and morphogenesis at various degrees. Accordingly, abnormal intracellular motility of organelles and vesicles has been observed, which directly results in altered cell morphogenesis (Wang et al., 2006; Gutierrez et al., 2009; Kato et al., 2010; Akkerman et al., 2012; Li et al., 2014).

Cotton is an important cash crop throughout the world, and its fibers are the primary raw material for the textile industry. The development of cotton fibers comprises four distinct, yet overlapping stages: fiber initiation, cell elongation, secondary cell wall (SCW) synthesis, and maturation (Haigler et al., 2012). There is a distinct rearrangement of the actin cytoskeleton during transition from fiber elongation to secondary wall deposition (Seagull, 1990; Wang et al., 2010). In line with the cytological observations, a variety of ABP encoding genes have been found to be preferentially expressed in developing fiber cells, such as those encoding actin deploymerizing factors (Wang et al., 2009), profilins (Wang et al., 2005; Wang et al., 2010; Bao et al., 2011), and LIM-domain proteins (Han et al., 2013; Li et al., 2013; Li et al., 2018). Moreover, studies of transgenic cotton suggest that some increases in F-actin abundance are beneficial for fiber quality improvement (Wang et al., 2009; Han et al., 2013), and formation of the higher actin cytoskeleton structure plays a determinant role in the progression of developmental phases of cotton fibers (Wang et al., 2010; Zhang et al., 2017). However, the detailed physiological roles of the actin cytoskeleton in cell and plant morphogenesis remain poorly understood.

In *G. hirsutum*, Ligon lintless-1 (Li_1_) is a monogenic dominant mutant that exhibits various abnormal morphological characteristics, including dwarf plants, twisted organs and extremely shortened fibers (Griffee and Ligon, 1929; Kohel, 1972). Therefore much work has been done to isolate the *Li* *_1_* gene (Karaca et al., 2002; Rong et al., 2005; Gilbert et al., 2013; Jiang et al., 2015; Thyssen et al., 2015). Recently, Thyssen et al. (2017) reported that the actin gene *Gh_D04G0865* is responsible for the Li_1_ mutants. However, the molecular mechanisms underlying the action of the *Li* *_1_* gene are largely unknown. In this independent study, we isolated the same gene using a map-based cloning approach. Here, we named it *GhLi* *_1_*, which harbors a missense mutation that causes the 65th glycine to be substituted with valine (G65V) in its gene product. Further analysis indicated that the G65V substitution in GhLi_1_ affected the nucleation and elongation processes during the F-actin assembly, which disturbs actin cytoskeleton organization in a dominant manner. We also showed that GhLi_1_ negatively regulates cotton plant morphology and fiber elongation by disordering the actin cytoskeleton-based processes such as intracellular transportation. Our results confirm and extend the current understanding of the role of the actin cytoskeleton in plant cell elongation.

## Materials and Methods

### Plant Materials and Growth Conditions

The Li_1_ mutants were provided by Dr. Kohel (USDA-ARS, College Station, TX, USA). It had been self-pollinated for at least six generations in our laboratory. Hai7124 is a commercial Sea-island *Verticillium*-resistant cultivar. Three BC_1_ populations and two F_2_ populations were developed from crosses between Li_1_ and Hai7124 between 2011 and 2016 (**Supplementary Table S1**). Petals, ovules, and fibers were collected from the adult Li_1_ mutants and wild-type (WT) plants. Roots, stems, and leaves were collected 15 days after germination (DAG) from seedlings grown in a growth chamber (16-h light/8-h dark, 28°C).

### Measurement of the Length of Root Elongation Zone and Cells From This Zone in 7 DAG Seedlings

The length of root elongation zone was determined by measuring the distance between the bottom of mature zone (characterized by root hairs) and the top of meristematic zone (V-shaped structure of apex) as described by Li et al. (2014). The length of cells from the root elongation zone was measured by the ImageJ software (https://imagej.nih.gov/ij/). Three-cm length root tips were incubated with FM4-64 staining buffer (5 mM) for 5 min at 25°C, and then observed under a LSM780 confocal laser microscope (Zeiss, Germany). The FM4-64 fluorescence was excited at 515 nm, with emission at 640 nm.

### Scanning Electron Microscopy (SEM) Analysis

Cotyledons, ovules, and fibers were fixed with 2.5% glutaraldehyde in phosphate buffered saline (PBS, pH 7.0) for 24 h and washed three times with PBS for 15 min at each step. They were postfixed with 1% OsO_4_ in PBS for 2 h, washed three times, then dehydrated by a graded series of ethano (30%, 50%, 70%, 80%, 90%, and 95%) for 15 min at each step, and finally dehydrated two times by absolute ethanol for 20 min. They were then coated with gold-palladium, and observed under a SEM (Hitachi Model SU-8010, Japan).

### Transmission Electron Microscopy (TEM) Analysis

Fibers were detached from the mature seeds and pre-treated as described in SEM analysis. After pre-treatment, samples were subjected to absolute acetone for 20 min, 1:1 mixture of acetone and Spurr resin for 1 h at room temperature, 1:3 mixture of acetone and Spurr resin for 3 h and to final Spurr resin for 24 h, and embedded in Spurr resin to heat at 70°C for 9 h. Six-µm sections were generated, stained by uranyl acetate and alkaline lead citrate for 10 min, and finally viewed using a TEM (Hitachi Model H-7650, Japan).

### Measurements of Length and Weight of Cotton Fibers

Bolls from the WT and Li_1_ plants were harvested at 10–50 days post anthesis (DPA) at 10 day intervals. Seeds were separated from each other using a boiling method (Schubert et al., 1973). After washing in flowing water, the straightened fibers were measured using a vernier caliper. Fibers were then separated from seeds and fully dried, before being weighed on an analytical balance. Fiber density on each seed was calculated as the ratio of weight to length.

### Determination of Cellulose Content in Cotton Fibers

Determination of the cellulose content in fibers was performed according to a method described previously (Zhang et al., 2017). Alcohol-insoluble fiber residues were treated with 2 M trifluoroacetic acid at 121°C for 90 min and centrifuged for 5 min. The pellets were re-suspended in 1-ml Updegraff reagent (acetic acid: nitric acid: water, 8:1:2) and incubated at 100°C for 30 min. After centrifugation, the residual materials were washed with water, 70% ethanol and acetone, in sequence, and then fully dried and weighed. Cellulose content was calculated as a percentage of the weight of the residual materials to that of the initial samples.

### Mapping of the *Li* *_1_* Gene

First, using three BC_1_ populations and microsatellite markers that were developed based on genome sequences of *G. raimondii* (Paterson et al., 2012), the *Li* *_1_* locus was mapped to a 1.088-cm region on Chromosome D04 (Liang, 2015). To further delimit the mapping interval, we developed two F_2_ populations and insertion-deletion (InDel) markers based on genome sequences of *G. hirsutum* and *G. barbadense* (Liu et al., 2015; Zhang et al., 2015). Finally, the *Li* *_1_* locus was delimited to a 630-kb interval on chromosome D04 of *G. hirsutum*. The cDNA and gDNA of 11 genes in this region were amplified from Li_1_ and WT plants, and compared by Clustal X 2.0 software (Larkin et al., 2007). A single-nucleotide polymorphism (SNP) marker was developed according to the base mutation in *Gh_D04G0865*. Primer sequences used for mapping and gene cloning are listed in **Supplementary Table S2**. A SMARTer RACE cDNA amplification kit (Clontech, USA) was used to isolate the 5’- and 3’-untranslated regions (UTRs) of *Gh_D04G0865*.

### Quantitative RT-PCR (qRT-PCR) Analysis

qRT-PCR analysis was performed on the ABI 7500 Real Time System (Applied Biosystems, USA). The amplification parameters were as follows: 95°C for 10 min, followed by 40 cycles at 95°C for 15 s, 58°C for 15 s, and 72°C for 15 s. The cotton *Histone 3* gene (*His3*, GenBank accession no. AF024716) was used as the internal control. Amplification efficiency and gene specificity of qRT-PCR primers (**Supplementary Table S2**) were tested by Sanger sequencing and polyacrylamide gel electrophoresis.

### Characterization of Actin Family Genes in *G. hirsutum*

The genomic database of *G. hirsutum* acc. TM-1 was downloaded from http://mascotton.njau.edu.cn/ (Zhang et al., 2015). Eight Arabidopsis actin protein sequences (McDowell et al., 1996) were used as queries to perform BLASTP search against the *G. hirsutum* protein sequence database. The identified actin genes were named as *GhACTs* based on their chromosomal location (**Supplementary Table S3**). ClustalX 2.0 software (Larkin et al., 2007) was used for the alignment of actin sequences from cotton and Arabidopsis. A phylogenetic tree was constructed by the Neighbor-Joining (NJ) method by MEGA v5.0 software and the reliability of interior branches was assessed with 1,000 bootstrap re-samplings (https://www.megasoftware.net/). The high-throughput RNA-sequencing data of *G. hirsutum* acc. TM-1 were employed from the accession code SRA: PRJNA248163 in the National Center for Biotechnology Information (https://www.ncbi.nlm.nih.gov/). Expression levels were calculated using the fragments per kilobase of exon model per million mapped reads (FPKM) method using Cufflinks software with default parameters (Trapnell et al., 2012). Heat map represent the spatio and temporal expression of *GhACTs* was drawing by Heml 1.0 software (Deng et al., 2014). Eight *GhACTs* were randomly chosen for qRT-PCR experiment, and a correlation analysis was conducted to test the consistency in gene expression between qRT-PCR and RNA-Seq methods. Primers used for qRT-PCR are listed in **Supplementary Table S2**.

### Virus Induced Gene Silencing (VIGS) Experiments in Cotton

Cotton VIGS assays were performed as described by Gao et al. (2011). Gene fragments were cloned into the pTRV2 vector and introduced into *Agrobacterium tumefaciens* strain GV3101. *Agrobacterium* cells were inoculated, harvested, and resuspended in infiltration medium (10-mM MgCl_2_, 10-mM MES, 200-mM acetosyringone), and adjusted to an OD_600_ of 1.5. Cell suspensions were incubated at 25°C for 3 h, and then *Agrobacterium* cultures carrying pTRV1 and pTRV2 or its derivatives were mixed at a 1:1 ratio. WT and Li_1_ seedlings (15 DAG) with fully expanded cotyledons and one true leaf were infiltrated by inserting the *Agrobacterium* suspension into the cotyledons. Primers used for vector construction and qRT-PCR analysis are listed in **Supplementary Table S2**.

### Observation of the Actin Cytoskeleton in Fiber and Root Cells

Observation of the actin cytoskeleton in fiber cells was performed as described by Seagull (1990). Fibers were incubated in PBS buffer containing 100-mM PIPES, 0.05% Triton X-100, 1-mM MgCl_2_, 3-mM DTT, 0.3-mM PMSF, 5-mM EGTA, 0.25% glutaraldehyde, and 0.66-µM AlexaFluor488-phalloidin for 10 min. The actin cytoskeleton in root cells was stained using the method described by Yang et al. (2011). Samples were incubated in PME buffer (100-mM PIPES, 5-mM MgSO_4_, and 10-mM EGTA, pH 6.9) containing 300-µM MBS, 1.5% glycerol, and 0.1% Triton X-100 for 30 min. Samples were rinsed twice with PME buffer and then fixed in PME buffer containing 2% paraformaldehyde for 30 min. Samples were incubated in the staining buffer (PME, 1.5% glycerol, 0.1% Triton X-100, and 0.66-µM AlexaFluor488-phalloidin) at 4°C overnight, washed twice with PME and mounted onto glass slides. Observation of F-actin was performed under a LSM780 confocal laser microscope (Zeiss, Germany). AlexaFluor488 fluorescence was excited at 488 nm with emission at 543 nm.

### Observation of Vesicles in Fiber and Root Cells

The distribution of vesicles in fiber and root cells was investigated according to the methods described by Zhao et al. (2010) and Li et al. (2014), respectively. Fibers and 1-cm root tips were respectively incubated with FM4-64 staining buffer (5 mM) for 10 min and 30 min at 25°C, and observed under a LSM780 confocal laser microscope (Zeiss, Germany). The FM4-64 fluorescence was excited at 515 nm, with emission at 640 nm.

### Immunofluorescence Analysis of CSC Distribution in Fiber Cells

The distribution of CSC in fiber cells was investigated according to the method described by Wang et al. (2010). Twenty DPA fibers were fixed with 4% paraformaldehyde and 0.1% glutaraldehyde in PME buffer containing 0.1% Triton X-100 and 0.3 M mannitol. After three washes in PME buffer, fibers were incubated with 1% cellulase R-10 and 0.1% pectolase Y-23 in PME buffer for 5 min. Fibers were washed once in PME buffer and twice in PBS (pH 7.0), and further incubated in PBS containing 1% BSA for 15 min. The fibers were then probed with the primary antibody (anti-CesA7; Agrisera, Sweden) and the corresponding secondary antibody (AlexaFluor594-conjugated anti-rabbit IgG; Abbkine, USA), washed twice with PBS and observed. The AlexaFluor594 fluorescence was excited at 588 nm with emission at 612 nm.

### Quantitative Analysis of Fluorescence Intensity

Quantification analysis of F-actin, vesicle, and CSC in fiber or root cells was conducted by ImageJ software (https://imagej.nih.gov/ij/) as described method by Zhang et al. (2011). Images belonging to the same comparison group were collected under the same condition, and then normalized to an equal grayscale. Sixty cells from six randomly selected visual fields were analyzed, and the region of interest size was 40 µm × 10 µm and 100 µm × 30 µm for fiber and root cell, respectively. The average pixel intensity were used to plot fluorescence intensity.

### Molecular Model of G-Actin and F-Actin

The multiple alignment of 75 actin protein sequences (the 1–120 N terminal amino acids were presented) from yeast, Arabidopsis, rice, maize, poplar, and upland cotton was performed by Clustal X2.0 software. The accession number of these proteins are listed in **Supplementary Tables S4** and **S5**. The molecular model of G-actin was constructed using SWISS-MODEL software (https://www.swissmodel.expasy.org/) using a previously reported G-actin structure, 3chw.1.A, as the template. The polymer structure 6anu.1.E was used as the template to construct an F-actin model, and the Swiss-PdbViewer software was used to simulate the structural changes caused by the G65V substitution (Guex et al., 2009).

### Recombinant Protein Production

The CDS of *GhLi* *_1_* and *Ghli* *_1_* were cloned into pET30a plasmids to construct *GhLi* *_1_*-6×His and *Ghli* *_1_*-6×His vectors, respectively. Recombinant proteins were expressed in *E. coli* strain BL21 (DE3) and purified using nickel-nitrilotriacetic acid resin. Purified GhLi_1_-6×His and Ghli_1_-6×His proteins were dissolved in Buffer A (5-mM Tris-HCl, pH 8.0, 0.2-mM CaCl_2_, 0.2-mM ATP, 0.1-mM imidazole and 0.5-mM DTT) to generate Ca-ATP-actins.

### Actin Nucleation Assay

Actin nucleation assays were conducted according to the methods described by Michelot et al. (2005). Mg-ATP-actin was prepared by incubation of Ca-ATP-actin with 1-mM EGTA and 0.1-mM MgCl_2_ for 2 min on ice. Monomeric rabbit skeletal muscle actin (2 µM; 10% pyrene-labeled) was incubated with different concentrations of GhLi_1_-6×His or Ghli_1_-6×His for 5 min at room temperature. Pyrene fluorescence was detected by an F-4600 fluorescence spectrophotometer (Hitachi, Japan) immediately after the addition of one-tenth volume of 10×KMEI buffer (500-mM KCl, 10-mM MgCl_2_, 10-mM EGTA, and 100-mM imidazole-HCl, pH 7.0). The fluorescence signal was detected every 50 s for a total of 20 min.

### Fluorescence Microscopy of Actin Filaments *in* *Vitro*

*In vitro* actin filaments were observed as described previously (Michelot et al., 2005). Ghli_1_-6×His and GhLi_1_-6×His proteins were mixed at different concentrations (2:0, 2:0.2, 0:2, in a unit of “µM”) in 1× KMEI buffer at 22°C for 5 or 20 min and labeled with an equimolar amount of rhodamine-phalloidin. The polymerized F-actin was diluted to 10 nM in fluorescence buffer (10-mM imidazole-HCl, pH 7.0, 50-mM KCl, 1-mM MgCl_2_, 100-mM DTT, 100-µg/ml glucose oxidase, 15 mg/ml glucose, 20 µg/ml catalase, and 0.5% methylcellulose). A dilute sample of 2 µl was applied to a 22 × 22-mm cover slip coated with poly-L-Lys (0.01%). Actin filaments were observed with an Axio Imager D2 microscope (Zeiss, Germany) equipped with a 60×, 1.42-numerical aperture oil objective. Images were collected with a Axiocam 503 color camera using ZEN 2010 software. After 5-min polymerization, the number of actin filaments were calculated to reflect actin nucleation capacity. Ten 50 × 50-µm regions were randomly selected from the images to calculate the number of actin filaments. The data was finally normalized to number per 1 mm^2^. After 20-min polymerization, the length of actin filaments were calculated to reflect actin elongation capacity. Ten 50 × 50-µm regions were randomly selected from the images to measure the length of actin filaments. The data represent means of each filament in each region. ImageJ software (https://imagej.nih.gov/ij/) were used in the measurement of filament number and length.

## Results

### Li_1_ Exhibits Defects in Cell Elongation

**Figure 1:**
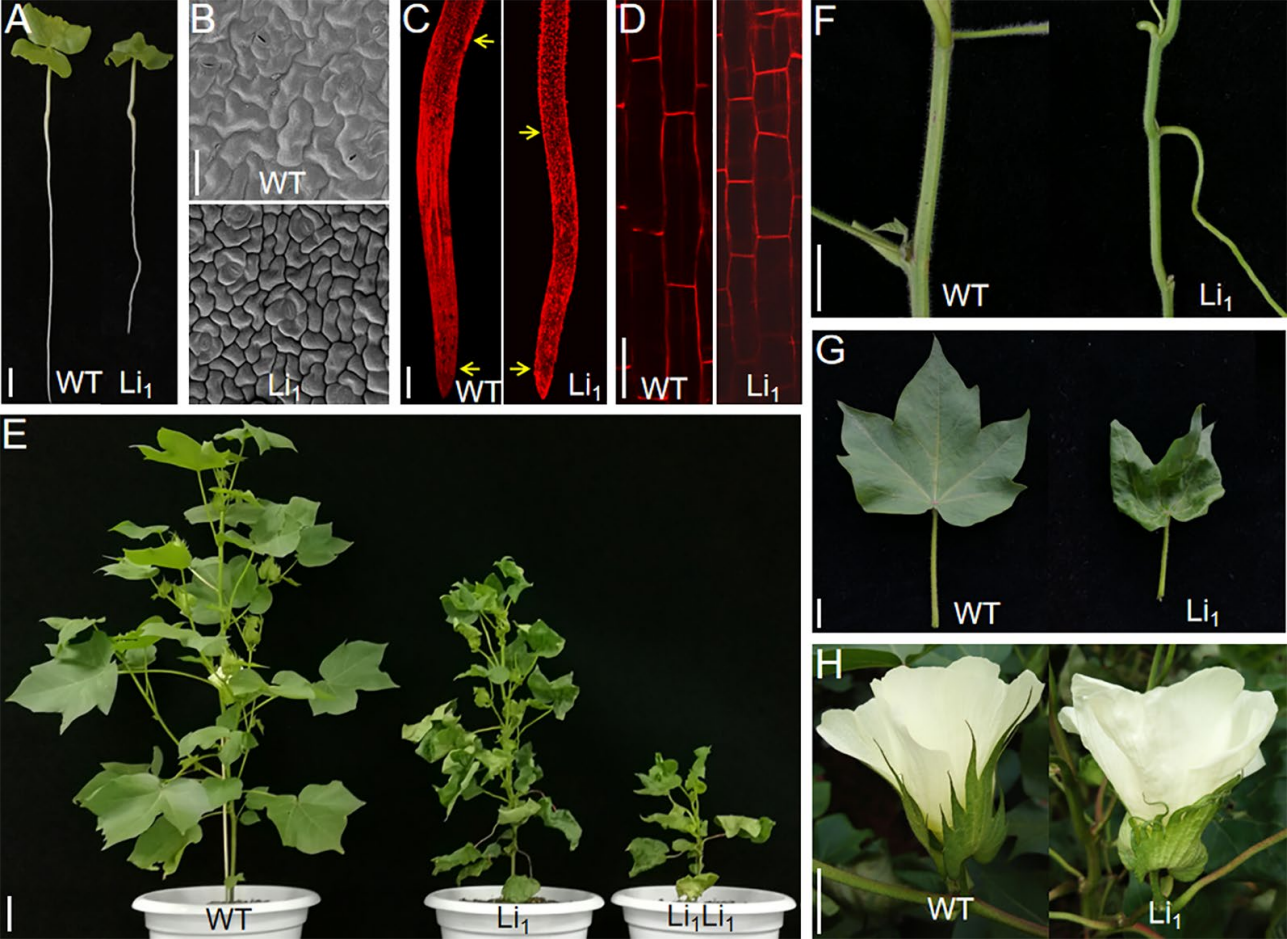
Phenotypic differences between WT and Li_1_. **(A)** WT and Li_1_ seedlings (7 DAG), showing reduced root growth and wrinkled cotyledons in the Li_1_. Bar = 2 cm. **(B)** SEM analysis of the cotyledon pavement cells of WT and Li_1_ seedlings (7 DAG), showing reduced surface area of the pavement cells in the Li_1_. Bar = 50 µm. **(C) **Root tips of WT and Li_1_ seedlings (7 DAG). Arrows indicate borders of the root elongation zone (between the bottom of mature zone and the top of meristematic zone), showing shorter length of the root elongating zone in the Li_1_. Bar = 0.1 cm. **(D**) Morphology of cortical cells in the root elongation zone of WT and Li_1_, showing shorter cell length in the Li_1_. Bar = 100 µm. **(E)** Compared with WT, the Li_1_ heterozygote and homozygote (Li_1_Li_1_) mutants exhibited semi and extremely dwarf plants, respectively. Bar = 5 cm. **(F**, **G**, **H)** Stems **(F)**, leaves **(G)**, and calyxes and petals **(H)** of WT and Li_1_ plants. Bars = 2 cm.

**Figure 2:**
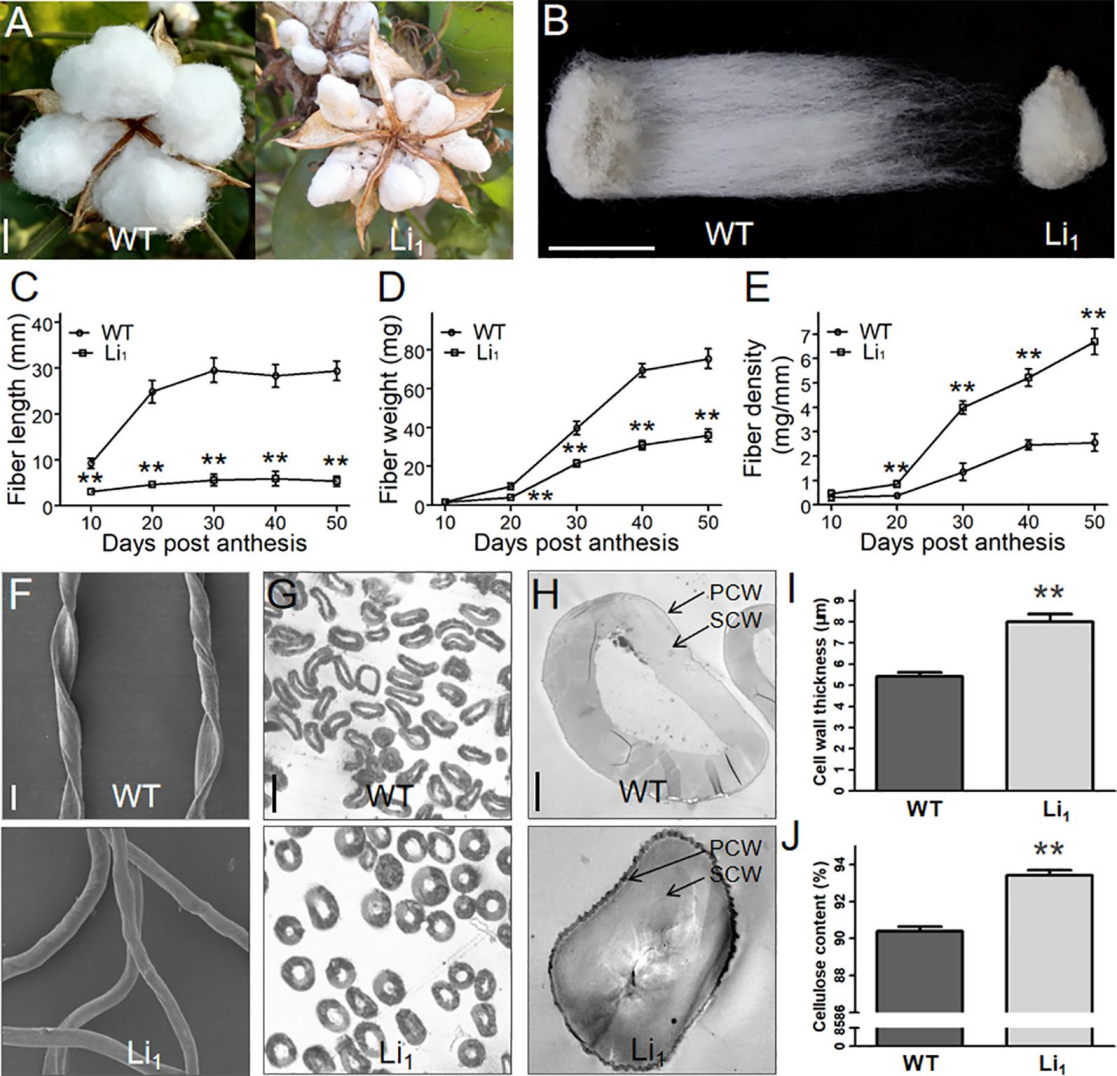
Comparison of the length, weight, density, and cell wall of WT and Li_1_ fiber cells. **(A**, **B)** Cracked bolls **(A)** and single seeds **(B)** of WT and Li_1_ showed extremely shortened fibers in the Li_1_ at maturity. Bars = 1 cm. (C, D, E) Measurement of the length **(C)**, weight **(D)** and density **(E)** of WT and Li_1_ fibers at different developmental points. **(F)** SEM analysis of mature fibers of WT and Li_1_. Bar = 20 µm. **(G)** Cross sections of mature fibers of WT and Li_1_. Bar = 20 µm. **(H)** Transmission electron microscopy images of mature fibers of WT and Li_1_ plants. PCW, primary cell wall; SCW, secondary cell wall. Bar = 5 µm. **(I)** Cell wall thickness of mature fibers of WT and Li_1_ plants. **(J)** Cellulose content of mature fibers of WT and Li_1_ plants. Data in **(C**, **D**, **E**, **I**, **J)** show means ± SD of measurements from 30 biological replicates, Student’s *t*-test: **P < 0.01.

Compared with the WT, the Li_1_ heterozygote showed reduced root growth and wrinkled cotyledons at the seedling stage (**Figure 1A** and **Table 1**), and dwarf plants, distorted stems, short internodes, wrinkled leaves, and petals (**Figures 1E–H**), and extremely shortened fibers at the later stages of development (**Figures 2A, B**). The Li_1_ homozygote showed low survival rate and much weaker growth than the Li_1_ heterozygote (**Figure 1E** and **Supplementary Table S1**). The more greatly affected phenotype of homozygote Li_1_ than heterozygote indicates that the phenotypic differences are caused by a semi-dominant gene or a dosage effect, in line with our previous report (Liu et al., 2010). Since the Li_1_ plants exhibited pleiotropic morphological changes, it is reasonable to speculate that the *Li* *_1_* gene is widely expressed in various organs/tissues. In addition, SEM analysis showed that the fiber development occurred at the same pace in the Li_1_ and WT between 0 and 2 DPA, whereas the Li_1_ fibers were obviously shorter than that of the WT on 3 DPA ovules (**Supplementary Figure S1**). Our observations are in agreement with those of previous studies (Karaca et al., 2002; Liang et al., 2015), suggesting that the *Li* *_1_* gene is not expressed until 3 DPA in developing fibers.

Given the smaller size of the Li_1_ mutants, we sought to compare the cellular morphology of cotyledons and roots in Li_1_ and WT plants. In the developing WT cotyledons, characteristic lobed and jigsaw-puzzle shaped pavement cells were found (**Figure 1B**). However, pavement cells in the Li_1_ plants had fewer lobes, and their surface area was significantly reduced in comparison to the WT (**Figure 1B** and Table 1). In the WT, pavement cells maintained a firm contact with their neighbors, whereas contiguous epidermal cells in the Li_1_ plants broke contact at their fringe, resulting in disconnected cells (**Figure 1B**). FM4-64 staining showed that the root elongation zone and cells from this zone were significantly shorter in Li_1_ seedlings than that in WT (**Figures 1C, D** and **Table 1**). These results suggest that polar growth and elongation of the Li_1_ cells was inhibited.

To gain more insights into the changes in developing Li_1_ fiber cells, we determined the length and weight of the Li_1_ and WT fibers at different developmental points. In the WT, fibers rapidly elongated between 10 and 20 DPA, and stopped at ∼30 DPA. Whereas the elongation of Li_1_ fibers nearly ceased at 10 DPA, and the Li_1_ fibers were significantly shorter than the WT (**Figure 2C**). Although the dry weights of both Li_1_ and WT fibers rapidly increased between 20 and 50 DPA (**Figure 2D**), the fiber density of Li_1_ plants during this period was significantly higher than that of WT (**Figure 2E**). SEM analysis showed that the WT fibers adopt a flat ribbon like structure with a continuous helix, while the Li_1_ fibers were shaped like circular tube and had no helix (**Figure 2F**). The increased density and altered shape of Li_1_ fibers prompted us to investigate their cell wall structure. Cross-sections analysis showed the cell wall (especially the SCW) of mature Li_1_ fibers had a much thicker appearance than that of the WT (**Figures 2G, H**). Statistical analysis showed that the thickness of the cell wall was significantly higher in the Li_1_ fiber cells (**Figure 2I**). Since the major component of mature cotton fiber cells is a SCW mainly consists of cellulose, the cellulose content of the mature fiber cells was measured. As shown in **Figure 2J**, the cellulose content of the Li_1_ mature fibers was significantly higher than that of the WT. Thus, more active cellulose synthesis may account for the thicker SCW, increased fiber density and non-helical fiber cell shape of the mutants.

**Table 1:** Comparison of phenotypic differences between WT and Li_1_ seedlings (7 DAG).

Name	WT	Li_1_
Length of the root	14.6 ± 1.8 cm	11.3 ± 1.3 cm**
Surface area of cotyledon pavement cells	1230 ± 151 µm^2^	462 ± 65 µm^2^**
Length of the root elongating zone	1.19 ± 0.16 cm	0.81 ± 0.11 cm**
Cell length in the root elongation zone	208.4 ± 23.1 µm	146.5 ± 14.0 µm**

Data represent the means ± SD from 30 biological replicates. Student’s t-test: **P < 0.01.

### Mapping of the *Li* *_1_* Gene

**Figure 3:**
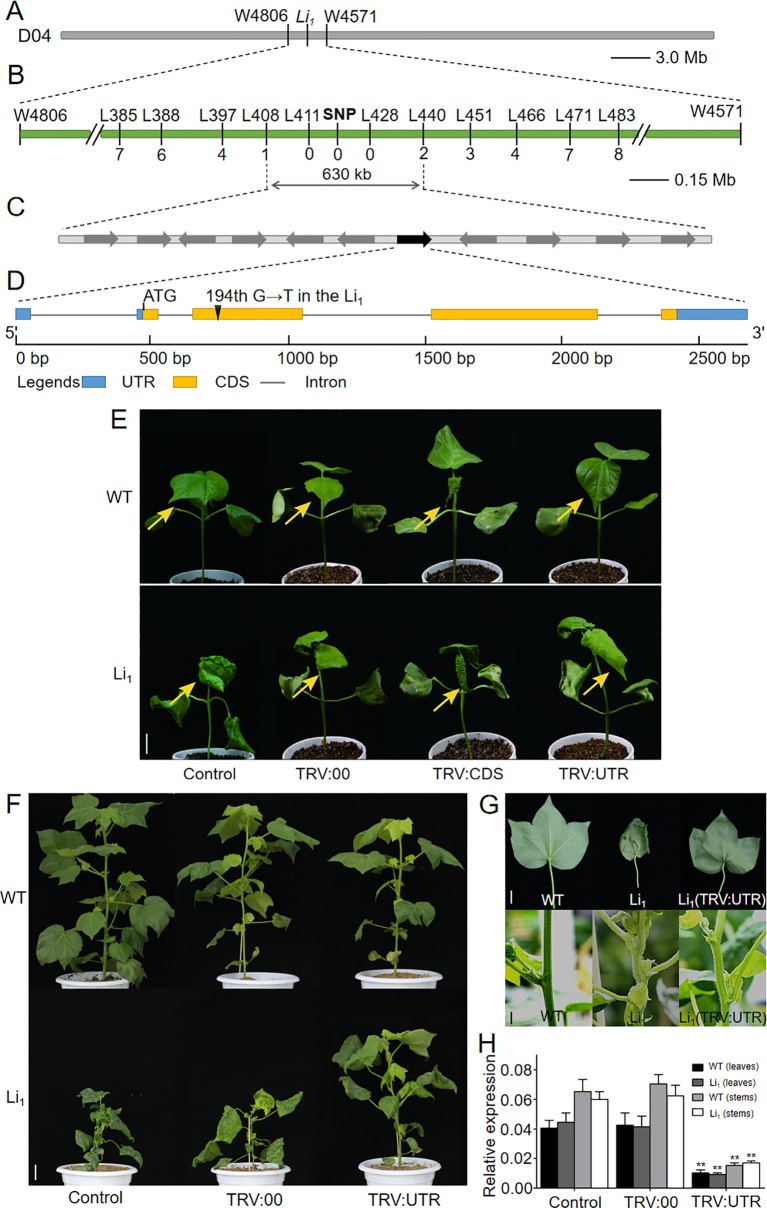
Map-based cloning and functional verification of the *Li* *_1_* gene. **(A)** The *Li* *_1_* locus was first mapped on chromosome D04 between the microsatellite markers W4806 and W4571 using three BC_1_ populations. **(B)** The *Li* *_1_* locus was further fine-mapped to a region between InDel markers L408 and L440 using two F_2_ populations, and a SNP marker designed by the base mutation in *Gh_D04G0865* was found to be co-segregated with the mutant phenotype. **(C)** The 630-kb mapping region includes eleven annotated genes, among which *Gh_D04G0865* (indicated by green box) was the candidate gene responsible for the Li_1_ mutants. **(D)** Gene structure of *Gh_D04G0865*. In the Li_1_, the 194th base of *Gh_D04G0865* had changed from “G” to “T”, resulting in the 65th amino acid residue being changed from glycine to valine (G65V) in the encoded actin. **(E)** Phenotypic changes were observed in the second true leaves (arrows indicated) 15 days after VIGS treatment. Bar = 2 cm. **(F)** The phenotypes of untreated (control), and pTRV2 (TRV:00) and pTRV2::UTR (TRV : UTR) treated WT and Li_1_ adult plants (90 day after VIGS treatment). Bar = 5 cm. **(G)** Phenotype of the leaves and stems of WT, Li_1_, and pTRV2::UTR treated Li_1_ plants. Bars = 2 cm. **(H)** Expression level of *Gh_D04G0865* in leaves and stems in control, and TRV:00 and TRV : UTR treated WT and Li_1_ adult plants. Data in **(H)** represent the means ± SD of three biological replicates, Student’s *t*-test: **P < 0.01.

To understand the mechanisms of *Li* *_1_* gene response to Li_1_ mutant phenotype, we isolated the causative gene using a map-based cloning approach. The *Li* *_1_* locus was first mapped on chromosome D04 between the microsatellite markers W4806 and W4571 using three BC_1_ populations, covering a 3.1 Mb interval (22.56–25.66 Mb) on chromosome D04 based on the genome sequence of *G. hirsutum* (**Figure 3** **A**; Liang, 2015; Zhang et al., 2015). Using two F_2_ populations and InDel markers, the *Li* *_1_* locus was further narrowed down to a region between L408 and L440, covering a 630-kb interval that covering 11 annotated genes (**Figures 3B, C**and **Supplementary Table S3**) according to the *G. hirsutum* genome (Zhang et al., 2015). Sequence analysis showed that the 194th base of *Gh_D04G0865* had changing from “G” to “T” in the Li_1_, resulting in the 65th amino acid residue being changed from glycine to valine (G65V) in the encoded actin (**Figure 3D**). Whereas the other ten genes had no sequence variation between the WT and Li_1_. The SNP marker designed by the base mutation in the CDS of *Gh_D04G0865* was found to be co-segregated with the Li_1_ phenotype (**Figure 3B**). The expression of annotated genes in the mapping region was determined in various organs and developing ovules (0, 3, 5 DPA) and fibers (10 DPA) from the WT and Li_1_ plants (**Supplementary Figure S2**). Eight genes didn’t conform to the broad spectrum expression feature of the *Li* *_1_* gene: *Gh_D04G0861*, *Gh_D04G0867*, *Gh_D04G0868*, and *Gh_D04G0869* did not express in all tested tissues; both *Gh_D04G0859* and *Gh_D04G0866* not in petals; *Gh_D04G0860* and *Gh_D04G0862* not in roots and petals, respectively. These genes didn’t conform to the broad spectrum expression feature of the *Li* *_1_* gene, so they were excluded from candidate genes. The expression level of *Gh_D04G0863* and *Gh_D04G0864* could not be distinguished due to their sequence similarity, and they expressed in all the tested tissues. The expression of *Gh_D04G0865* could be found in all tissues except for the 0 DPA ovules. Statistical analysis showed the expression level of these genes were not significantly changed in Li_1_ tissues in comparison to that in WT. However, interestingly, spatiotemporal expression pattern of *Gh_D04G0865* is highly consistent with the phenotypic features of Li_1_, including the mutant phenotype in various tissues and the onset time (3 DPA) of abnormal development in fibers (**Figure 1** and **Supplementary Figures S1** and **S2**). Therefore, it was supposed that the actin gene *Gh_D04G0865* was the most likely candidate gene underlying the *Li* *_1_* locus.

### Actin Family Genes in *G. hirsutum*

In higher plants, actin is typically encoded by a multigene family, and one of the most important feature of actin genes is that they share high sequence similarity (Šlajcherová et al., 2012). Hence, understanding of the members, sequence characters, and expression patterns of the actin family genes in *G. hirsutum* is essential prior to the functional studies of *Gh_D04G0865*. A BLASTP search against the *G. hirsutum* genome identified 37 actin genes that dispersed on 20 of the 26 chromosomes. They were named as *GhACT1A*∼*GhACT17A* and *GhACT1D*∼*GhACT17D* according to their chromosomal location (**Supplementary Figure S3** and **Supplementary Table S4**). Arabidopsis actin family genes were the most thoroughly studied actin family genes in higher plants, they were grouped into vegetative and reproductive classes based on their phylogenetic relationship and expression pattern (McDowell et al., 1996). Phylogenetic analysis showed cotton actin genes were also divided into two classes A and B that clustered together with the reproductive and vegetative Arabidopsis actin genes, respectively (**Supplementary Figure S4**). Subsequent divergence divided class A and B into three and two subclasses, respectively. Gene and protein sequence similarity of *GhACTs* with each other were higher than 80.17% and 90.64%, respectively. *Gh_D04G0865* (named as *GhACT9D* in this nomenclature) shares higher than 81.74% CDS similarity with other *GhACTs* (**Supplementary Figure S5**). Transcriptomic data showed the expression pattern of *GhACTs* were in concordance with their phylogenetic divergence. With a few exceptions, most *GhACTs* in the group A were specially expressed in the reproductive organs such as stamens and developing ovules, while most *GhACTs* in the group B were expressed in almost all organs (**Supplementary Figure S6**). Eight *GhACTs* were randomly selected for qRT-PCR analysis, and their relative expression level were highly correlated with the transcriptomic data, indicating both of the two methods were credible in detection of gene expression level (**Supplementary Figure S7**). Expression pattern of *GhACTs* indicated that many members were likely to be simultaneously expressed in the given organs/tissues, and each gene product has the potential to participate in actin assembly to form hetero-polymer F-actins (Meagher et al., 1999).

### Functional Verification of the *Li* *_1_* Gene

Recently, Thyssen et al. (2017) reported that *Gh_D04G0865*, the same gene as we isolated, is responsible for the Li_1_ mutants. To verify that this gene is the causal gene for *Li* *_1_*, these researchers constructed a pTRV2 vector comprising its 781–1134-bp CDS (shares higher than 81.59% sequence similarity with other *GhACTs*) and 127 bp 3’-UTR, and VIGS experiments were performed in WT plants. The resultant decreased expression of *Gh_D04G0865* and the abnormal phenotypes of VIGS treated plants suggested that this gene is responsible for the Li_1_ mutants. However, we have different opinions on the design of this experiment. Firstly, the VIGS vector constructed by Thyssen et al. (2017) may not be appropriate for specifically silencing the expression of the target actin gene. In plants, VIGS can reduce the expression of endogenous genes that are homologous to the inserted fragment in the VIGS vector *via* RNA-mediated defense (RMD) mechanisms (Baulcombe, 1999). RMD recognizes the complementary RNA sequence by base pairing, thus unintended off-target genes may also be silenced if they share high sequence similarity with the inserted fragment, and the interpretation of the observed phenotypic changes may be consequently obscured (Senthilkumar and Mysore, 2011). In fact, this phenomenon has long been attracted the attention of researchers (Burch-Smith et al., 2004; Xu et al., 2006; Fantini et al., 2013; Kumar, 2014). To specifically known down the expression of an actin gene, *GhACT1*, in cotton, researchers have used its gene-specific 3’-UTR fragment for RNAi vector construction (Li et al., 2005). However, a conserved fragment could simultaneously knockdown the expression of other members of a gene family or other genes that share high sequence similarity with the trigger sequence (Ramachandran et al., 2000; Burch-Smith et al., 2004; Kandoth et al., 2007). Our present analysis of the actin family genes in *G. hirsutum* showed that the CDS of *GhACTs* share more than 80% sequence similarity with each other (**Supplementary Figure S5**). This is high enough for efficient gene silencing (Holzberg et al., 2002; Zhai et al., 2016). Secondly, the use of receptor plants in the VIGS experiment by Thyssen et al. (2017) is not suitable. Genetic study has proved that the Li_1_ mutant was caused by a semi-dominant gene, thus the “gain-of-function” gene *Li* *_1_* is required for the formation of the mutant phenotype in Li_1_. To verify the function of *Li* *_1_*, phenotypic changes in Li_1_ plants after silencing of the dominant gene *Li* *_1_* should be examined, since phenotypic changes in the WT plants after the reduced expression of the recessive gene cannot represent the function of *Li* *_1_*. This principle is supported by many previous RNAi and VIGS studies, such as those in rice (Huang et al., 2009; Jiao et al., 2010; Tang et al., 2011; Li et al., 2015) and cotton (Chang et al., 2016; Si et al., 2018).

To investigate whether the mutated actin gene *Gh_D04G0865* is the gene underlying the *Li* *_1_* locus, we firstly attempted to develop transgenic lines that overexpress the mutated *Gh_D04G0865* driven by the 35S promoter in WT cotton line W0 (Wu et al., 2008). However, we failed to generate positive plant with the expressed target gene, probably due to the detrimental effects on cell elongation of the mutated *Gh_D04G0865*. To investigate whether the mutated *Gh_D04G0865* is responsible for the Li_1_ mutant, and at the same time to verify our different opinions with Thyssen et al. (2017), we performed two groups of VIGS experiments. One fragment corresponding to 730–1121-bp CDS (shares higher than 81.22% sequence similarity with other *GhACTs*) and another corresponding to 1–297-bp 3’UTR of *Gh_D04G0865* were separately cloned into pTRV2 vector, generating pTRV2::CDS (TRV : CDS) and pTRV2::UTR (TRV : UTR) constructs. Li_1_ and WT seedlings were used as receptors to observe their phenotypic changes after VIGS treatment. Fifteen days after inoculation, the newly grown leaves were used for phenotypic observation since the morphology of older leaves had already formed before VIGS treatment. Leaves from all TRV : UTR treated WT plants had little change in comparison to that from untreated and TRV:00 (empty vector for control) treated WT plants (**Figure 3E**), probably due to the functional redundancy of actin genes as reported in Arabidopsis (for example: *act2*, *act4*, and *act7* T-DNA mutants showed little phenotype changes; Gilliland et al., 1998). Compared with untreated and TRV:00 treated Li_1_ plants, more flatten leaves were observed in 55 out of 60 TRV : UTR treated plants (**Figure 3E**). In contrast, very seriously deformed leaves were produced from 55 out of 60 TRV : CDS treated Li_1_, and 57 out of 60 TRV : CDS treated WT plants in TRV : CDS treated Li_1_ and WT plants, and this phenotype was radically different with untreated Li_1_ leaves (**Figure 3E**). Moreover, viability of these plants became too weak to produce the third true leaf. qRT-PCR was used to determine the expression level of nine-leaf-expression *GhACTs* as indicated by the transcriptomic data (**Supplementary Figures S6** and **S8**). As we expected, only *Gh_D04G0865* was significantly silenced in TRV : UTR treated Li_1_ and WT plants. In contrast, TRV : CDS significantly reduced the expression level of all of the tested *GhACTs* in Li_1_ and WT plants. These results demonstrated that this VIGS phenotype could not be used to explain the function of the targeted gene. Phenotypic changes in the VIGS experiment were more obvious at 90 days after treatment: TRV : UTR treated WT plants had little difference with untreated and TRV:00 treated plants; TRV : UTR treated Li_1_ plants exhibited relieved mutant phenotype characterized by taller plants (**Figure 3F**), more flatten leaves and straighter stems than that from two groups of control plants (**Figure 3G**). Expression level of *Gh_D04G0865* in the leaves and stems from TRV : UTR treated plants were significantly decreased (**Figure 3H**). These results suggested that the mutated *Gh_D04G0865* expression was tightly linked to the degree performance of Li_1_ plants and confirmed that the mutated *Gh_D04G0865* was responsible for the Li_1_ mutant. Given the dominant inherited feature of the Li_1_ mutants, *Gh_D04G0865* was named *Ghli* *_1_* and *GhLi* *_1_* for the wild and mutant types, respectively.

### Changes in Actin Cytoskeleton and Vesicle Distribution in Elongating Cells

**Figure 4:**
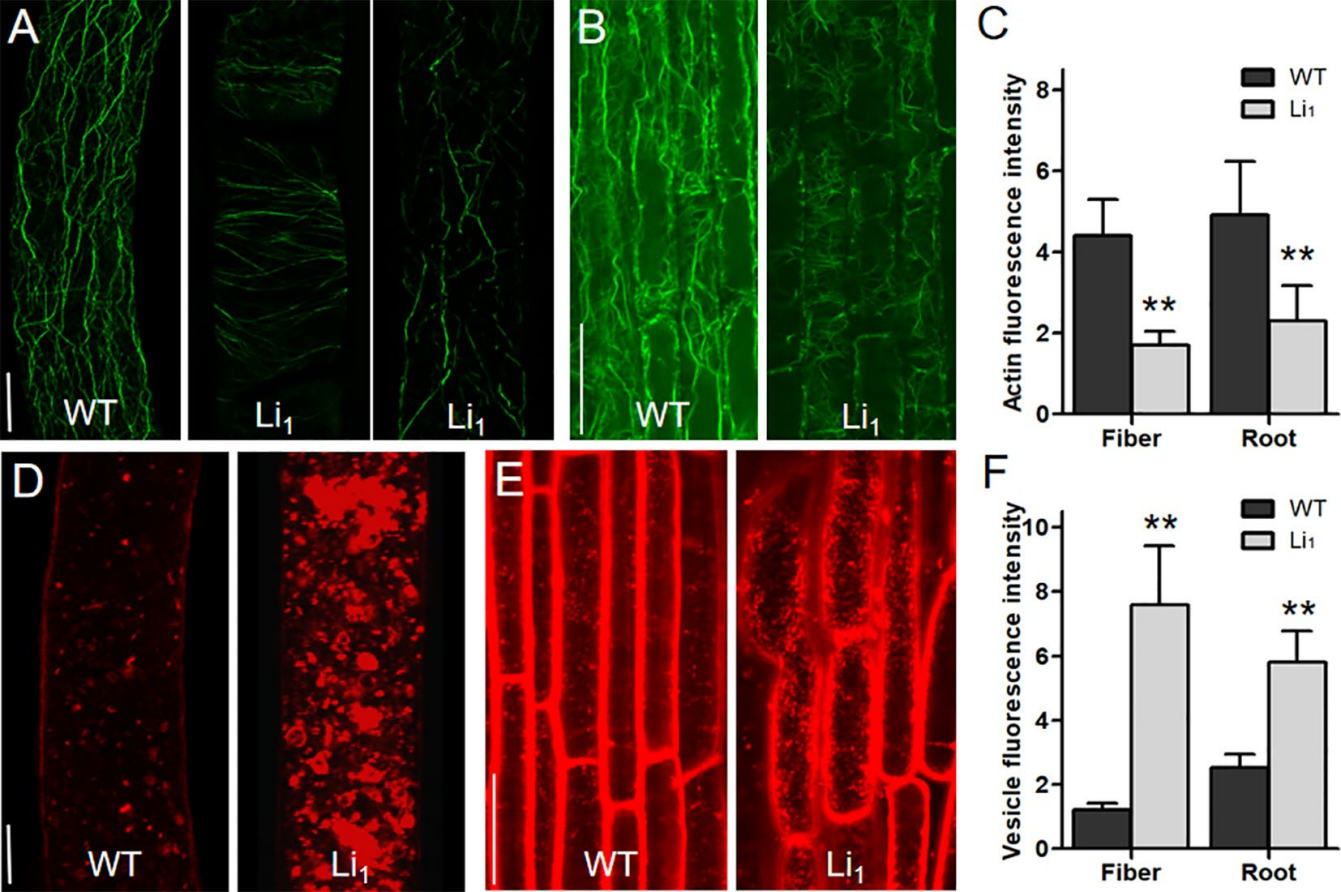
Distribution of F-actin and vesicles in elongating fiber and root cells. **(A)** F-actin organization in 10 DPA fiber cells of WT and Li_1_ plants. Bar = 10 µm. **(B)** F-actin organization in cortical cells from the root elongation zone of WT and Li_1_ plants. Bar = 100 µm. **(C)** Determination of the fluorescence intensity representing the amount of F-actin. **(D)** Vesicle distribution in 10 DPA fiber cells of WT and Li_1_ plants. Bar = 10 µm. **(E)** Vesicle distribution in cells from the root elongation zone of WT and Li_1_ plants. Bar = 100 µm. (F) Determination of the fluorescence intensity representing vesicle abundance. Data in **(C**, **F)** represent means ± SD of 60 biological replicates (60 cells), Student’s *t*-test: **P < 0.01.

Although it is clear that *GhLi* *_1_* encodes a mutated actin, the mechanisms by which the G65V substituted actin affects the organization of the actin cytoskeleton in elongating cells remains to be explored. In 10 DPA fibers, which undergo fast elongation, many fine F-actin cables were formed, and further arranged longitudinally to the axis of the fiber cell in the WT (**Figure 4A**). However, the F-actin cables in 10 DPA Li_1_ fibers were arranged horizontally or obliquely, and their distribution was obviously sparser than that of the WT. In some cases, F-actin cables in the Li_1_ fiber cells were fragmented (**Figure 4A**). The same differences in the organization of the F-actin cytoskeleton between the Li_1_ mutants and the WT were observed in cortical cells from the root elongation zone (**Figure 4B**). A quantitative measurement of the fluorescence intensity of the confocal images showed that the amount of F-actin in the Li_1_ elongating root and fiber cells was significantly lower than that in the WT (**Figure 4C**).

The actin cytoskeleton has been found to serve as tracks for intracellular transportation (Gutierrez et al., 2009). To investigate whether the intracellular transportation in the Li_1_ cells is affected, the distribution of vesicles in elongating fiber and root cells was observed. FM4-64 staining showed that vesicles were distributed evenly in the WT fiber cells; however, massive vesicles aggregated into patches were observed in the Li_1_ fiber cells (**Figure 4D**). Similarly, more labeled vesicles were present in the Li_1_ cells from root elongation zone than that in the WT (**Figure 4E**). Measurement of the fluorescence intensity showed significantly more vesicles in the Li_1_ elongating root and fiber cells than in the WT (**Figure 4F**). These observations suggest that intracellular transportation was disturbed in the Li_1_ elongating cells.

### Changes in the Distribution of the Actin Cytoskeleton, Vesicles, and CSC in Fiber Cells During SCW Biosynthesis

The more thickened SCW and higher cellulose content of Li_1_ fibers compared to WT fibers (**Figures 2G–J**) offers an ideal model for investigating the role of the actin cytoskeleton in SCW biosynthesis. We therefore examined whether the distribution of the actin cytoskeleton, vesicles, and CSC were affected in Li_1_ fiber cells at 20 DPA, when they undergo active SCW biosynthesis. As shown in **Supplementary Figure S9A**, the F-actin cables in the WT fiber cells became thick and straight; while fewer thick F-actin cables were formed, and those that did were fragmented and randomly arranged in the Li_1_ fiber cells. As in elongating fibers, vesicles in the Li_1_ fiber cells were aggregated into patches during SCW biosynthesis, a distribution dramatically different to that in corresponding WT fiber cells (**Supplementary Figure S9B**). To determine the CSC distribution in the Li_1_ and WT fibers, the commercial antibody anti-CesA4 was used for immunostaining experiments (Gardiner et al., 2003). As shown in **Supplementary Figure S9C**, the distribution of CSC was obviously increased in the Li_1_ fibers in comparison to the WT. Quantitative analysis showed the amount of F-actin was significantly reduced, while the number of vesicles and CSC were significantly increased in the Li_1_ fiber cells (**Supplementary Figure S9D–F**). Based on these results, we propose that the disturbed intracellular transportation and excessive distribution of CSC at the PM is the major reasons for the increased content of cellulose and thickened SCW of Li_1_ fiber cells.

### Effects of G65V Substitution on Actin Polymerization

**Figure 5:**
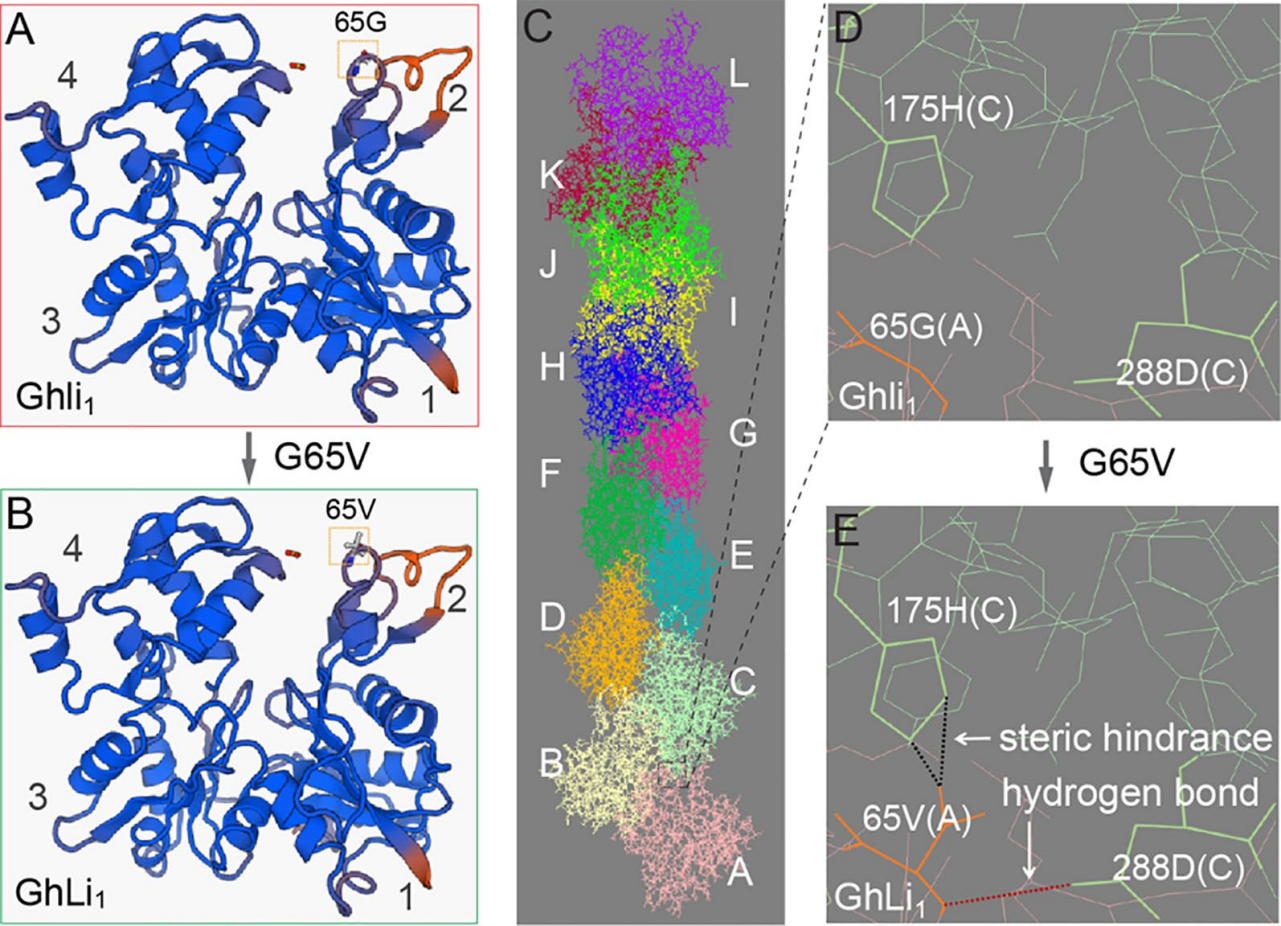
Molecular model prediction of the effect of G65V substitution in GhLi_1_. **(A**, **B)** Monomer structures of Ghli_1_ **(A)** and GhLi_1_ **(B)** were produced using SWISS-MODEL software with 3chw.1.A as the template. **(C)** The structure of an F-actin polymerized by Ghli_1_ was constructed using 6anu.1.E as the template; different monomer molecules **(A**–**I) **are colored distinctly. **(D**, **E)** Simulation of G65V substitution in GhLi_1_ showing that a steric hindrance (indicated by black dotted line) and a H-bond (indicated by red dotted line) emerged between the 65V in molecule A and 175H and 288D in molecule C, respectively. The side chain of 65V in molecule A, and 175H and 288D in molecule C are highlighted in bold.

Amino acid substitution in a protein as conserved as actin may dramatically affect its function, thus we investigated the influence of the G65V substitution in GhLi_1_. An extensive protein sequence alignment showed that the 65G was highly conserved in actin (**Supplementary Figure S10** and **Supplementary Table S5**), indicating that it may be necessary for actin’s basic function. The 65G is located on the surface-exposed region of subdomain 2, also known as a junction among G-actins in an F-actin (**Figures 5** **A–C**; Holmes et al., 1990). Simulation of G65V substitution in the F-actin molecule gave rise to a steric hindrance and a hydrogen bond between the 65V in molecule A and the 175H and 288D in molecule C, respectively (**Figures 5D, E**). Holmes et al. (1990) reported the atomic structure of F-actin and proposed that the hydrophobic “plug-pocket” interaction is essential for F-actin stabilization. According to the structural correspondence between the protein sequences of GhLi_1_ and the rabbit skeletal muscle actin used in the “Holmes model”, the occurrence of “plug-pocket” interactions in cotton actin can be depicted as shown in **Figure 5C**. In a normal F-actin, eight residues (positions 42–47, 65, and 66) of molecule A and six residues (positions 168, 171, 173, 175, 287, and 291) of molecule C form a hydrophobic pocket. At the same time, a hydrophobic loop (residues 266–275) of molecule B in the opposing strand generates a hydrophobic plug, which inserts into the hydrophobic pocket to bring about a strong three-bodied G-actin interaction. Moreover, some salt bridges are formed near this interaction zone to stabilize the F-actin structure, including one between the 41R of molecule A and the 288D of molecule C. According to our model, the steric hindrance and hydrogen bond caused by G65V substitution may affect the three-bodied G-actin interaction by changing the structure of the hydrophobic pocket and influencing the formation of the salt bridge, leading to instability of the F-actin.

**Figure 6:**
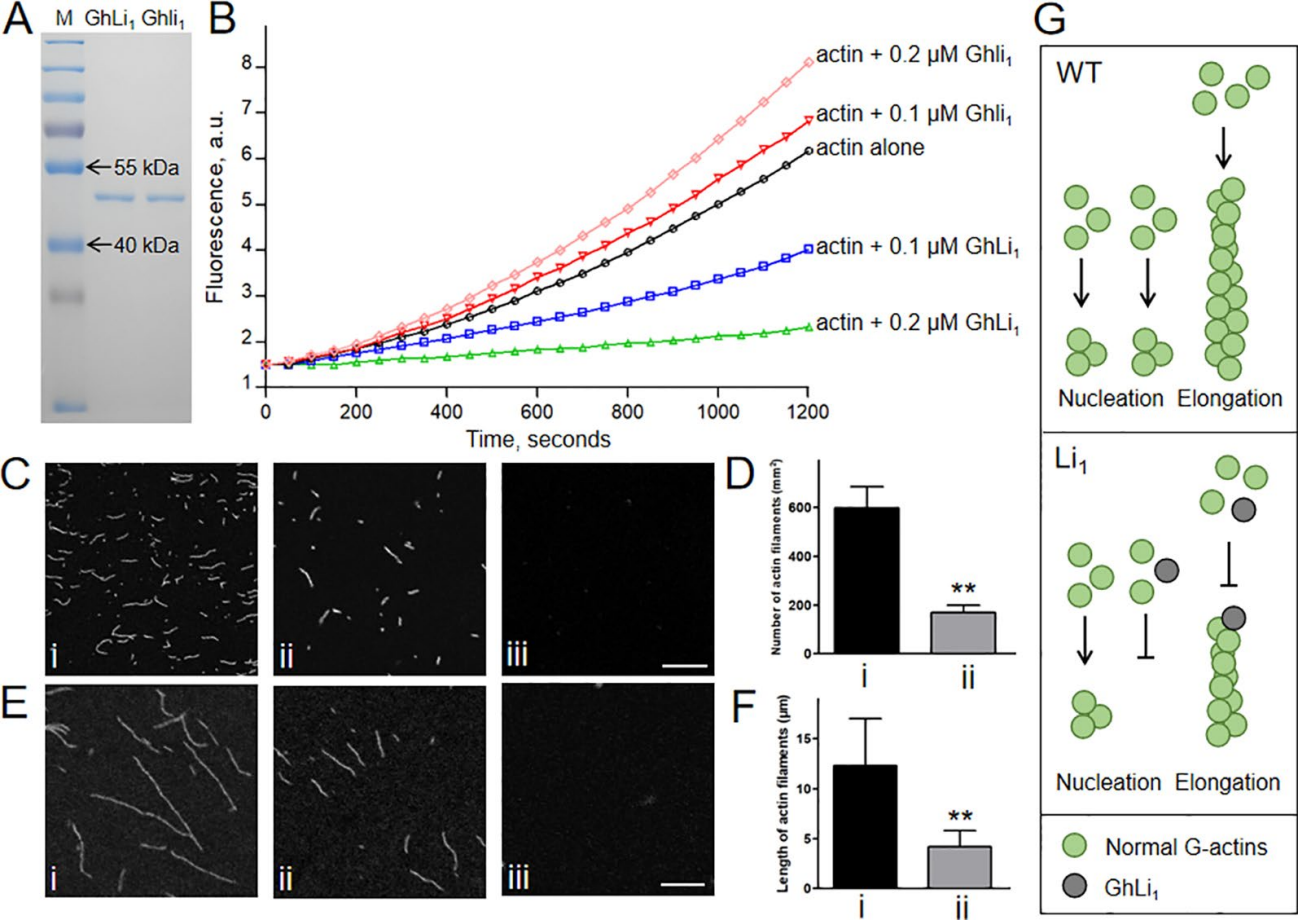
Determination of the polymerization capability of Ghli_1_ and GhLi_1_. **(A)** Purified 6×His tag fused GhLi_1_ and Ghli_1_ proteins were analyzed by SDS-PAGE and stained with Coomassie Brilliant BlueR 250. The protein marker (M) was loaded in the left lane. **(B)** Time course of actin polymerization in the presence of GhLi_1_ or Ghli_1_ monitored by pyrene fluorescence. The detailed data represent means, SD, and statistical analysis were listed in **Supplementary Table 6**. Different concentrations of GhLi_1_ or Ghli_1_ were added to 2 µM of 10% pyrene-labeled actin before initiation of polymerization. a.u. absorbance units. **(C**–**F)** 2 µM Ghli_1_ (i), mixture of 2 µM Ghli_1_ and 0.2 µM GhLi_1_ (ii) and 2 µM GhLi_1_ (iii) were polymerized in 1×KMEI buffer for 5 or 20 min, and visualized under fluorescence microscopy. After 5-min polymerization, actin filaments were observed **(C)** and the number of actin filaments per 1 mm^2^ of the visual field was calculated **(D)**. After 20-min polymerization, actin filaments were observed **(E)** and the mean length of actin filaments were determined **(F)**. Bars = 10 µm. Data in **(D**, **F)** represent the means ± SD of three biological replicates, Student’s *t*-test: **P < 0.01. **(G)** Proposed model for interpreting the effects of GhLi_1_ in F-actin dynamics. In WT, G-actins nucleate and elongate to bring about long F-actins; whereas in Li_1_, the existence of GhLi_1_ could disrupt F-actin assembly by inhibiting nucleation and elongation.

We next produced 6×His-tag fused GhLi_1_ and Ghli_1_ proteins to investigate their functional differences through *in vitro* biochemical assays (**Figure 6A**). To generate a new F-actin, three G-actin molecules must interact to create a seed, which is the rate-limiting step during spontaneous F-actin assembly (Pollard and Cooper, 1986). We performed *in vitro* analysis using the recombinant GhLi_1_ and Ghli_1_ proteins to test their nucleation ability. Actin monomers (10% pyrene labeled) from rabbit skeletal muscle were incubated with different concentrations of GhLi_1_ and Ghli_1_, and the actin assembly status was monitored by pyrene fluorescence measurement. As shown in **Figure 6B** and **Supplementary Table S6**, Ghli_1_ decreased the initial lag of the actin polymerization curve in a dose-dependent manner, which is indicative of their active roles in actin nucleation (Michelot et al., 2005). In contrast, the initial lag of the actin polymerization curve was increased as the increase of GhLi_1_ concentration, indicating that GhLi_1_ disturbs actin nucleation. The final amplitude of pyrene fluorescence, which is directly proportional to the actin filament concentration, was dramatically decreased with the addition of GhLi_1_, suggesting that actin polymerization was disturbed by this mutated actin.

To further investigate the polymerization capability of the recombinant GhLi_1_ and Ghli_1_ proteins, we observed actin filaments under fluorescence microscope. After 5-min polymerization, Ghli_1_ produced a large number of short actin filaments, whereas there were significantly fewer actin filaments produced from the mixture of Ghli_1_ and GhLi_1_, and GhLi_1_ alone did not produce any actin filaments under the same conditions (**Figures 6C, D**). After 20-min polymerization, Ghli_1_ produced long actin filaments, whereas actin filaments from the mixture of Ghli_1_ and GhLi_1_ were significantly shorter, and no actin filaments were produced by GhLi_1_ (**Figures 6E, F**). These results further confirm that GhLi_1_ disturbs nucleation and elongation of F-actin. Thus, it is fully reasonable to infer that the polymerization status in tissues with GhLi_1_ expression in the Li_1_ differs to that in the WT. We propose that, in the Li_1_ cells, GhLi_1_ could partake in, but disturbed, actin’s physical interactions (**Figure 6G**). When GhLi_1_ was involved in nucleation, normal seeds could not be formed. When GhLi_1_ was added onto the elongating end of an F-actin molecule by chance, it inhibited further polymerization by creating a “dead end”.

## Discussion

The actin cytoskeleton plays important roles in almost all cellular processes (Staiger et al., 2000; Blanchoin et al., 2010). Mutations in actin cause a range of malformed morphologies due to specific molecular changes that often disturb cytoskeleton functions (Feng and Marston, 2009; Kato et al., 2010). In this study, we report the isolation and characterization of *GhLi* *_1_*, which encodes a mutated actin with a G65V substitution in its gene product, from the Li_1_ mutants. Our results demonstrate that GhLi_1_ disorders actin cytoskeleton organization and intracellular transportation, and consequently alters the morphogenesis of cotton plants and cells.

### The Actin Cytoskeleton Plays Crucial Roles in Cell Elongation and the SCW Biosynthesis in Cotton

Actin cytoskeleton has been reported to essential for plant morphology. Previous studies had showed that disorganized actin cytoskeleton caused by pharmacological perturbation, mutation, or misexpression of actin and actin cytoskeleton-associated genes inhibited cell growth and altered the plant architecture (Kato et al., 2010; Wu et al., 2015). Cotton is a mainstay of the global economy and is prized for its excellent natural fiber properties. In this study, we showed that the dominant mutated actin gene *GhLi* *_1_* disorganized actin cytoskeleton, disrupts proper cell elongation, and resulted in various twisted organs. Thus, this report establishes that the mutation of a single actin can cause dramatic morphogenetic defects in cotton. Many studies have found that the actin cytoskeleton regulates cotton fiber development. Reduction in F-actin abundance inhibits fiber elongation (Seagull, 1990; Li et al., 2005), while thicker and more abundant F-actin cables promote fiber length and strength (Wang et al., 2009; Wang et al., 2010; Zhang et al., 2017). In this study, we found that the F-actin cables in Li_1_ were disorganized and fragmented (**Figure 4** and **Supplementary Figure S9**), which resulted in shortened and thickened fiber cells (**Figure 2**). Our findings provide solid genetic evidence of the pivotal roles of the actin cytoskeleton in regulating cotton fiber development.

During cell growth, vesicles containing cell wall matrix materials are delivered to expansion sites and fuse with the PM to deposit their contents to the cell wall (Ketelaar et al., 2003). Previous studies proposed that longitudinally oriented actin cables were the primary tracks for organelle movement (Gutierrez et al., 2009; Kato et al., 2010; Akkerman et al., 2012). During the fast elongation stage, cotton fibers undergo enormous polar growth, while massive cellulose molecules are synthesized during SCW deposition stage (Haigler et al., 2012). Thus, active cellular transport must take place in fiber cells during these development stages. It is conceivable that the reduction in longitudinal actin cables in the Li_1_ cells leads to abnormal vesicle transportation. Indeed, our cytological observations showed that vesicles were aggregated into patches, and more CSCs were inserted into the PM of Li_1_ cells than that of WT, which inhibited cell elongation and caused excessive cellulose deposition (**Figure 4** and **Supplementary Figure S9**). Based on these results, we hypotheses that F-actin may acts as a track for vesicle movement thus regulating elongation and SCW deposition in fiber cells.

### G65V Substitution in Actin Disturbs F-Actin Assembly

Actin is one of the most ancient proteins that is essential for the survival of eukaryotes. In accordance with actin’s high evolutionary conservation, most amino acid residues are critical for actin’s function. Over the past few decades, hundreds of actin mutants, responsible for various morphological changes, have been identified in both uni- and multi-cellular organisms (Ishiguro and Kobayashi, 1996; Feng and Marston, 2009; Kato et al., 2010). Actin mutants in human are the most thoroughly studied because they are causal for genetic diseases (Pierick et al., 2014; Marston, 2018). At the molecular level, these mutated actins were unable to fold properly to form functional actin, were incapable of polymerization, or had changes in their ability to interact with ABPs (Costa et al., 2004; Feng and Marston, 2009). With the availability of abundant information for actin mutants, a framework for genotype-phenotype correlation could be built and used for molecular diagnosis of human diseases (Marston, 2018), and also probably for the characterization of abnormal developments of animals and plants in view of the functional conservation of actin cytoskeleton. Prediction on the possible effects of each mutation based on its structural location and our knowledge of actin structure-function relationships is an efficient approach in understanding of their molecular mechanisms.

According to the Holmes model, the hydrophobic “plug-pocket” structure constitutes a three-bodied interaction that is essential for F-actin stability (Holmes et al., 1990). Many amino acid substitutions in, or near, this region exhibit different effects, for example I64N, T66I, G268C, D286G, and D291V in human skeletal actin ACTA1 exhibited reduced or completely lost polymerization capability, and resulted in various congenital myopathies (Feng and Marston, 2009). G273D in yeast actin ACT1 give rise to abnormal cell growth, probably because the amino acid substitution affects the hydrophobic “plug-pocket” interaction (Ishiguro and Kobayashi, 1996), and similar structural changes were proposed in E272K in Arabidopsis ACT8 (Kato et al., 2010). Position 65 in GhLi_1_, which is structurally identical to position 63 in the rabbit actin used by Holmes et al. (1990), participates in the hydrophobic “pocket” formation. Mutation in this position is novel and different from previously reported actin mutants. According to our model, the steric hindrance and hydrogen bond caused by G65V substitution may affect the three-bodied interaction by changing the hydrophobic pocket structure and influencing the formation of the salt bridge, which could be unfavorable for F-actin assembly (**Figure 5**; Holmes et al., 1990). Our biochemical assays demonstrated that GhLi_1_ was completely lost polymerization capability. In addition, it also inhibited nucleation and elongation activities normally occurred in F-actin assembly process (**Figure 6**). Thus, G65V substitution may converts GhLi_1_ from a normal linker to a “disturbing or capping protein”. Our results are in agreement with previous studies on actin mutants, and provide a new genotype-phenotype example of deciphering the process of abnormal development in plants. This model may also be useful for other eukaryotes.

## Data Availability Statement

The datasets generated for this study are available on request to the corresponding author.

## Author Contributions

TZ conceptualized the research program. TZ and YH designed the experiments and coordinated the project. YS, WL, WS, HF, and ZS conducted map-based cloning and functional analysis of the *Li* *_1_* gene. YS and JC developed the molecular markers. YS, YH, and TZ analyzed all of the data and wrote the paper.

## Funding

This study was financially supported in part by grants from the National Research and Development Project of Transgenic Crops of China (2015ZX08009-003) to YH, the earmarked fund for the China Agriculture Research System, and the Distinguished Discipline Support Program of Zhejiang University to TZ.

## Conflict of Interest

The authors declare that the research was conducted in the absence of any commercial or financial relationships that could be construed as a potential conflict of interest.
