# Supplementary material for: G65V Substitution in Actin Disturbs Polymerization Leading to Inhibited Cell Elongation in Cotton
Source: Front Plant Sci. 2019 Nov 15;10:1486. doi: 10.3389/fpls.2019.01486 (PMC6873290; doi:10.3389/fpls.2019.01486)
Supplement: Supplementary file 1 [file DataSheet_1.docx]

Supplementary Material

**
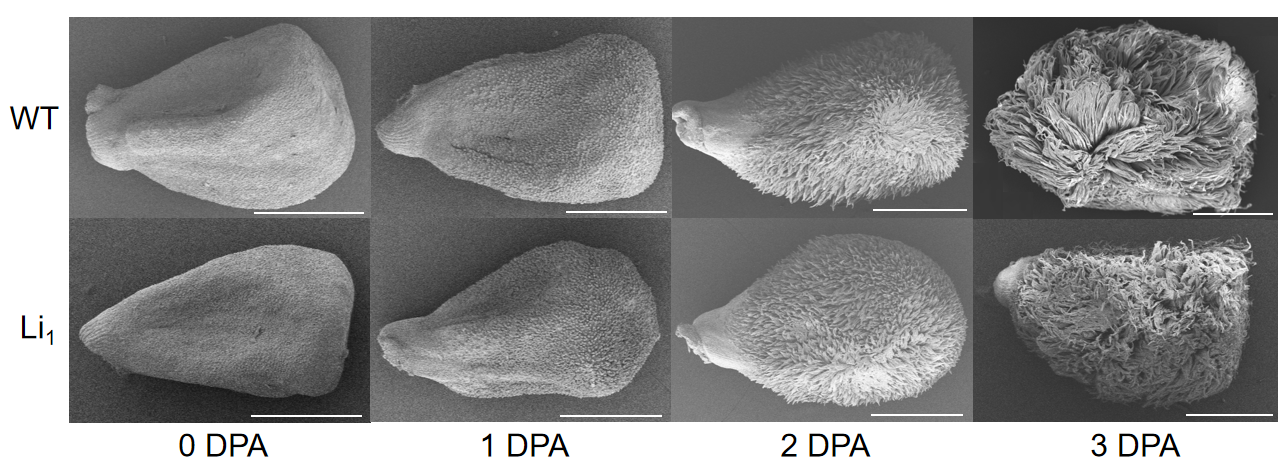
**

**FIGURE S1** SEM analysis of developing ovules and fibers of WT and Li_1_ at different developmental time-points. The fiber development occurred at the same pace in the Li_1_ and WT between 0 and 2 DPA, whereas the Li_1_ fibers were obviously shorter than that of the WT on 3 DPA ovules. Bars = 0.5 mm in all panels.


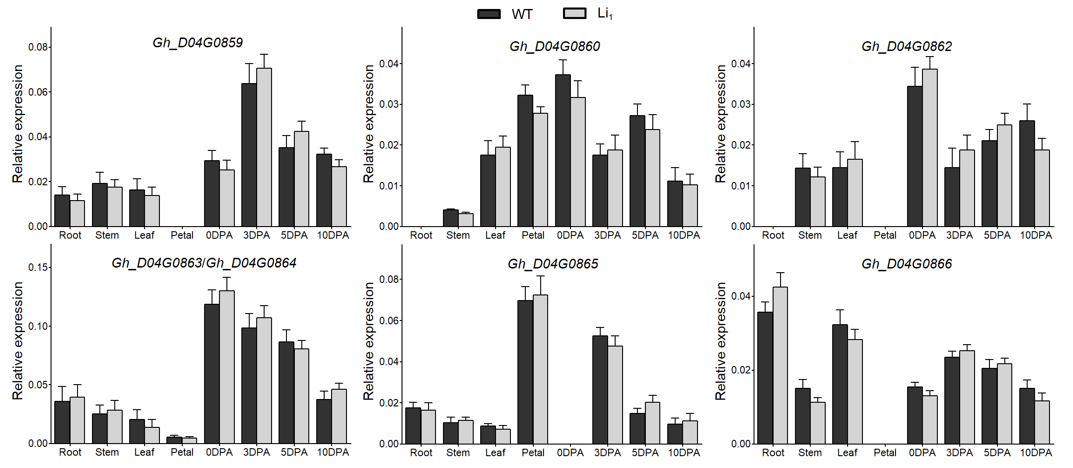


**FIGURE S2** Expression profiles of eleven annotated genes in the mapping region. The expression level of annotated genes in the mapping region in various organs and developing ovules (0, 3,5 DPA) and fibers (10 DPA) were determined by qRT-PCR. Data represent the means ± SD of three biological replicates.


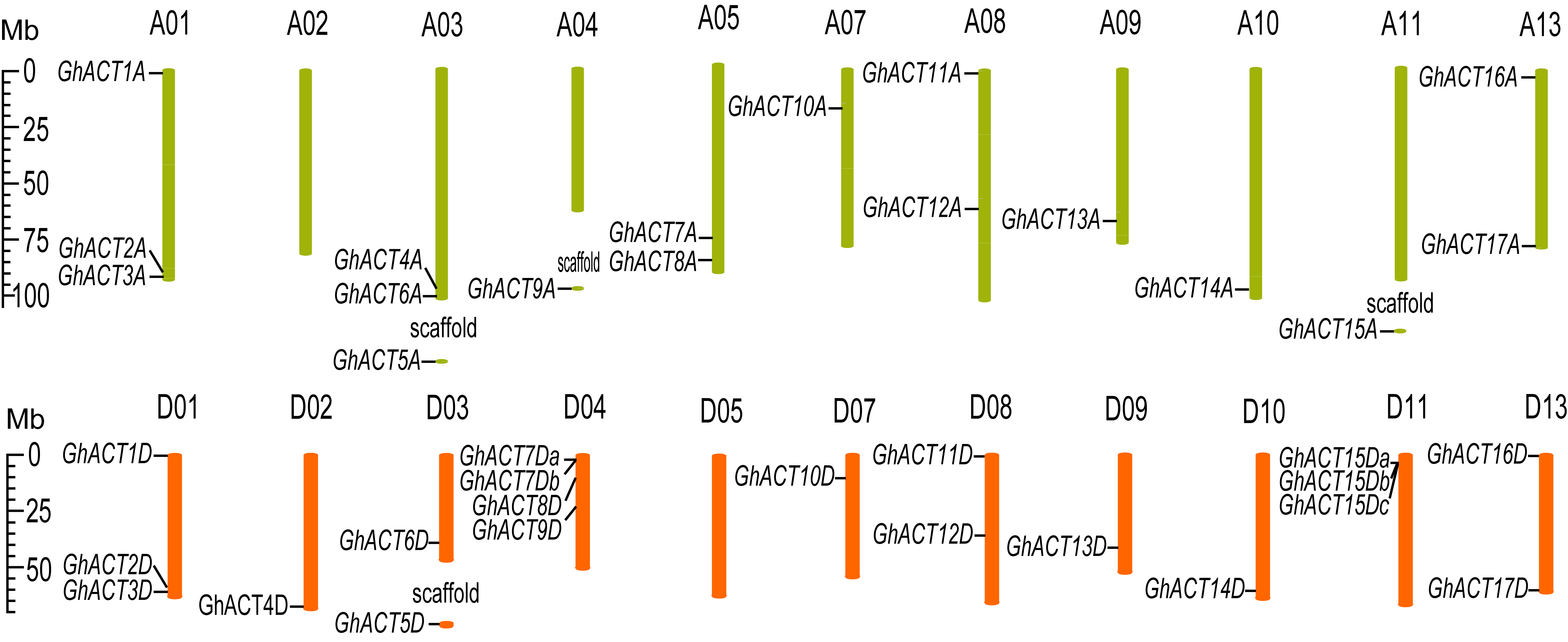


**FIGURE S3** Chromosomal distribution of *GhACTs*. The chromosome numbers was shown on the top of each bar. Green and orange bars represent physical maps of A and D subgenome, respectively. Lines on each bar indicate the location of *GhACTs*. The scale is in megabases (Mb).


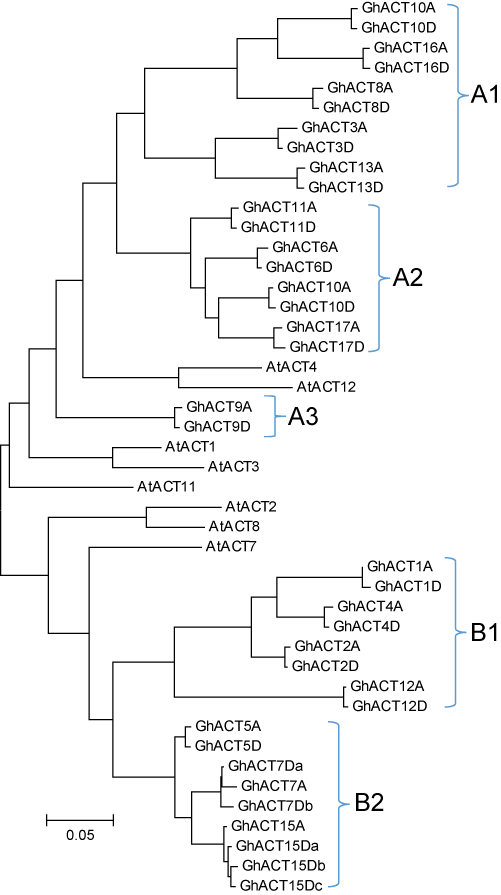


**FIGURE S4** Phylogenetic tree of actins from *G. hirsutum* and Arabidopsis**.** The amino acid sequences of eight Arabidopsis actins (AtACTs) and 37 GhACTs were aligned by ClustalX 2.0. The phylogenetic tree was constructed using MEGA 5.0 software by the Neighbour-Joining (NJ) method with 1,000 bootstrap replicates. The scale bar represents the number of amino acid substitutions per site.


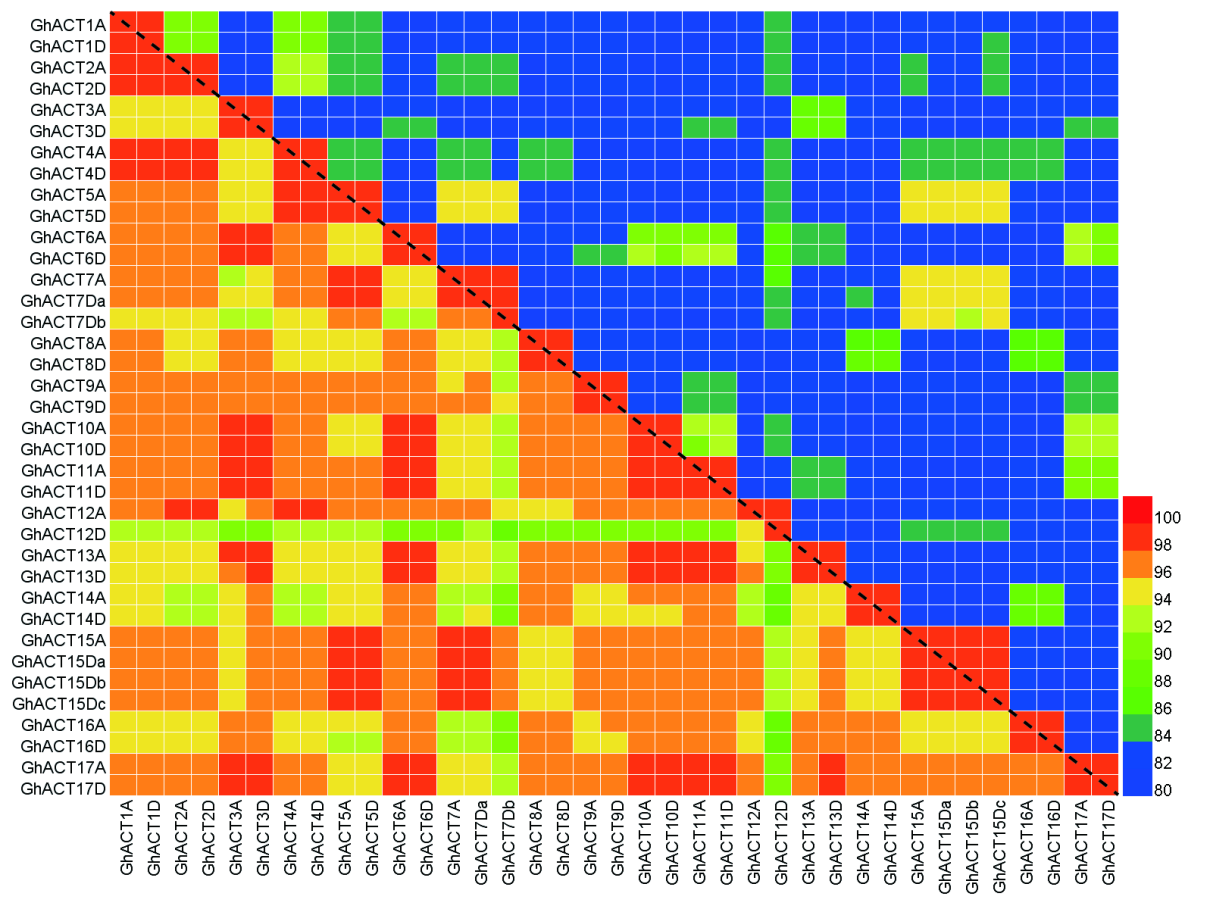


**FIGURE S5** Heat map representation of gene and protein sequence similarities of *GhACTs*. Data above and below the diagonal represents gene and protein sequences similarity, respectively. Color bar on the right indicate percent of similarity values.


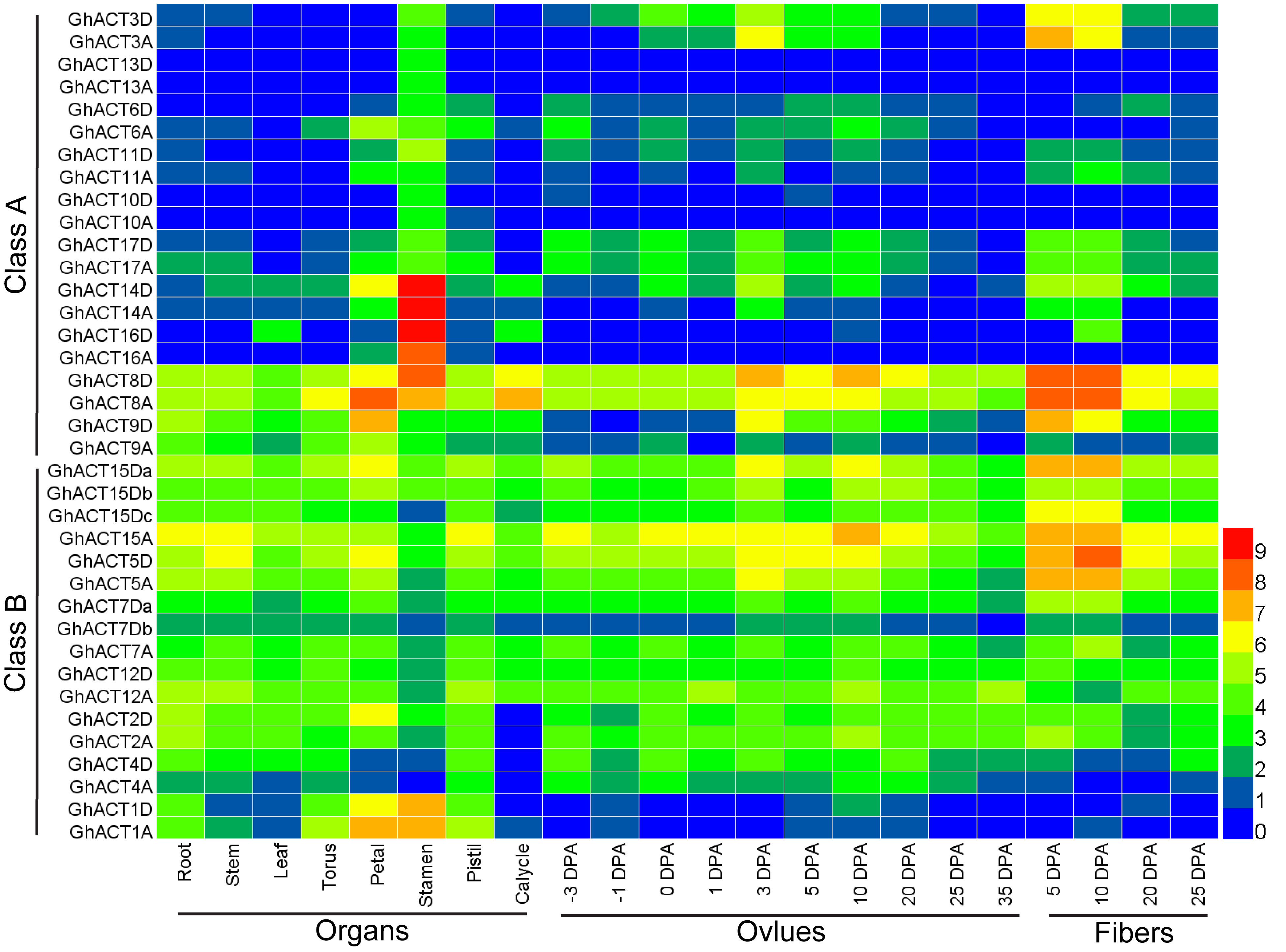


**FIGURE S6** Heat map representation of the expression patterns of *GhACTs* in different tissues. The heat map shows the RNA-Seq analysis of the spatio-temporal expression pattern of *GhACTs*. The tissues used for expression profiling analysis are indicated at the bottom. Genes were shown on the left of the expression bars. Scale bars on the bottom right of each heat map represent log_2_(RPKM + 1).


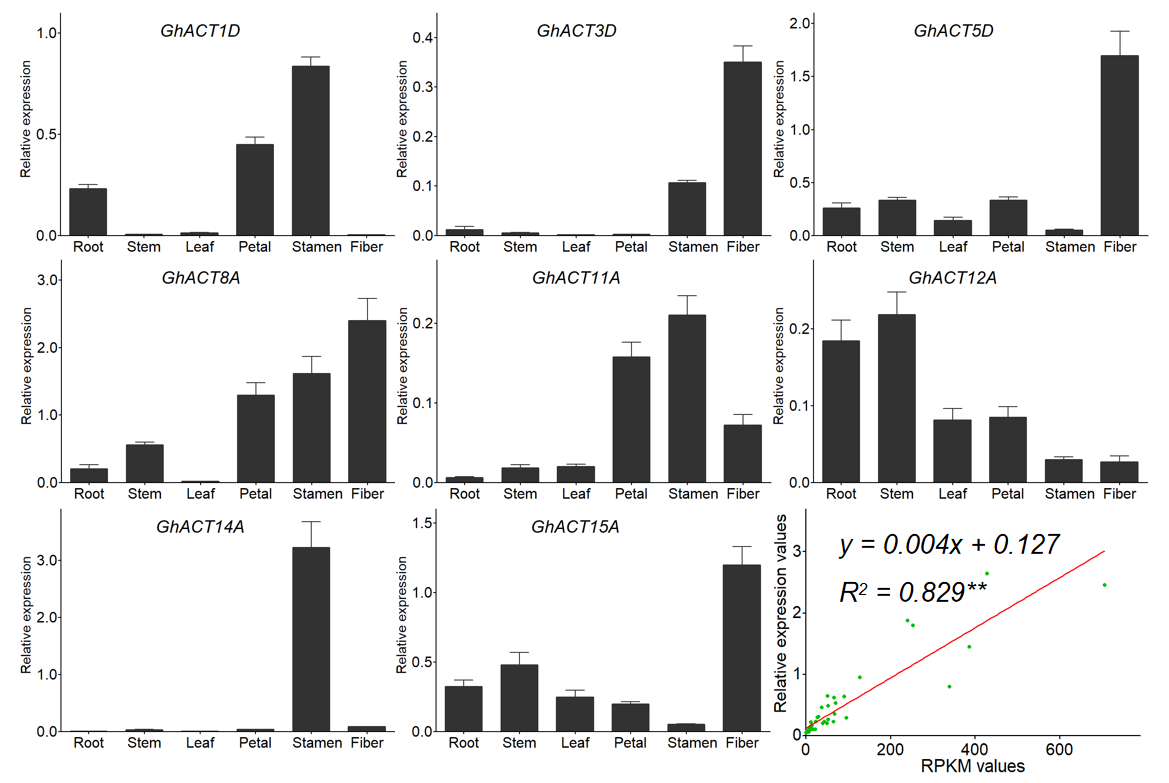


**FIGURE S7** qRT-PCR analysis of eight *GhACTs* in different tissues. Data represent the means ± SD of three biological replicate experiments. Correlation analysis between gene expression levels obtained from RNA-Seq and qRT-PCR was shown in the lower right corner figure. Student’s *t*-test: ***P* < 0.01.


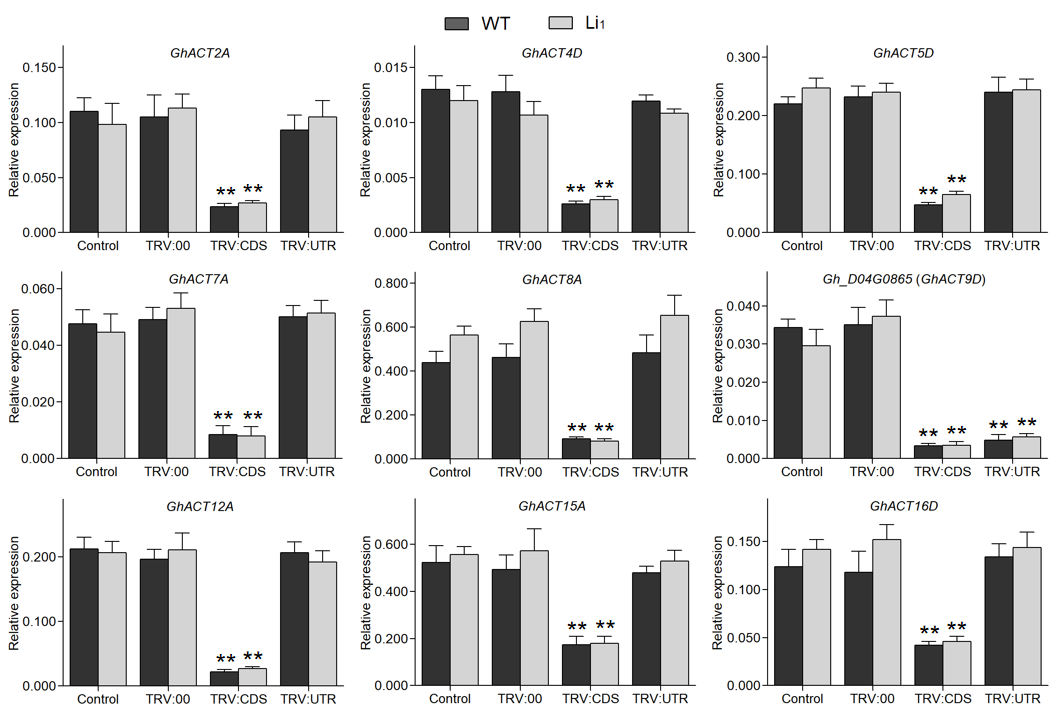


**FIGURE S8** Expression levels of *GhACTs* in VIGS treated seedlings. Fifteen days after VIGS treatment, the second true leaves (arrows indicated in Figure 3E) were used for qRT-PCR analysis. Data represent the means ± SD of three biological replicates, Student’s *t*-test: ***P* < 0.01.

**
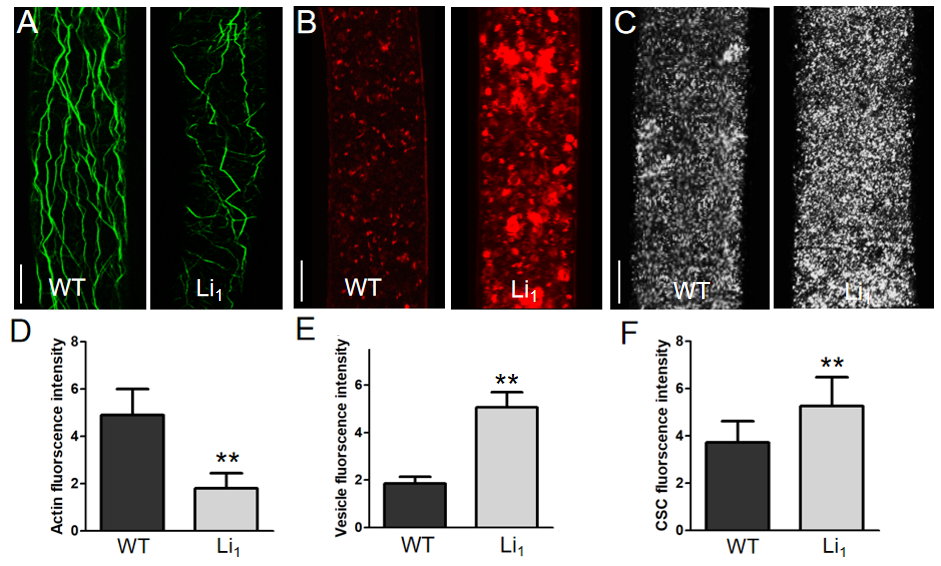
**

**FIGURE S9** Distribution of F-actin, vesicles and CSCs in fiber cells at SCW biosynthesis stage. (A-C) Confocal images of F-actin (A), vesicles (B) and CSC (C) in 20 DPA fiber cells of WT and Li_1_. Bars = 10 µm. **(D-F)** Determination of the fluorescence intensity representing of the amount of F-actin **(D)**, vesicles **(E)** and CSC **(F)**. Data represent means ± SD of 60 biological replicates (60 cells), Student’s *t*-test: ***P* < 0.01.


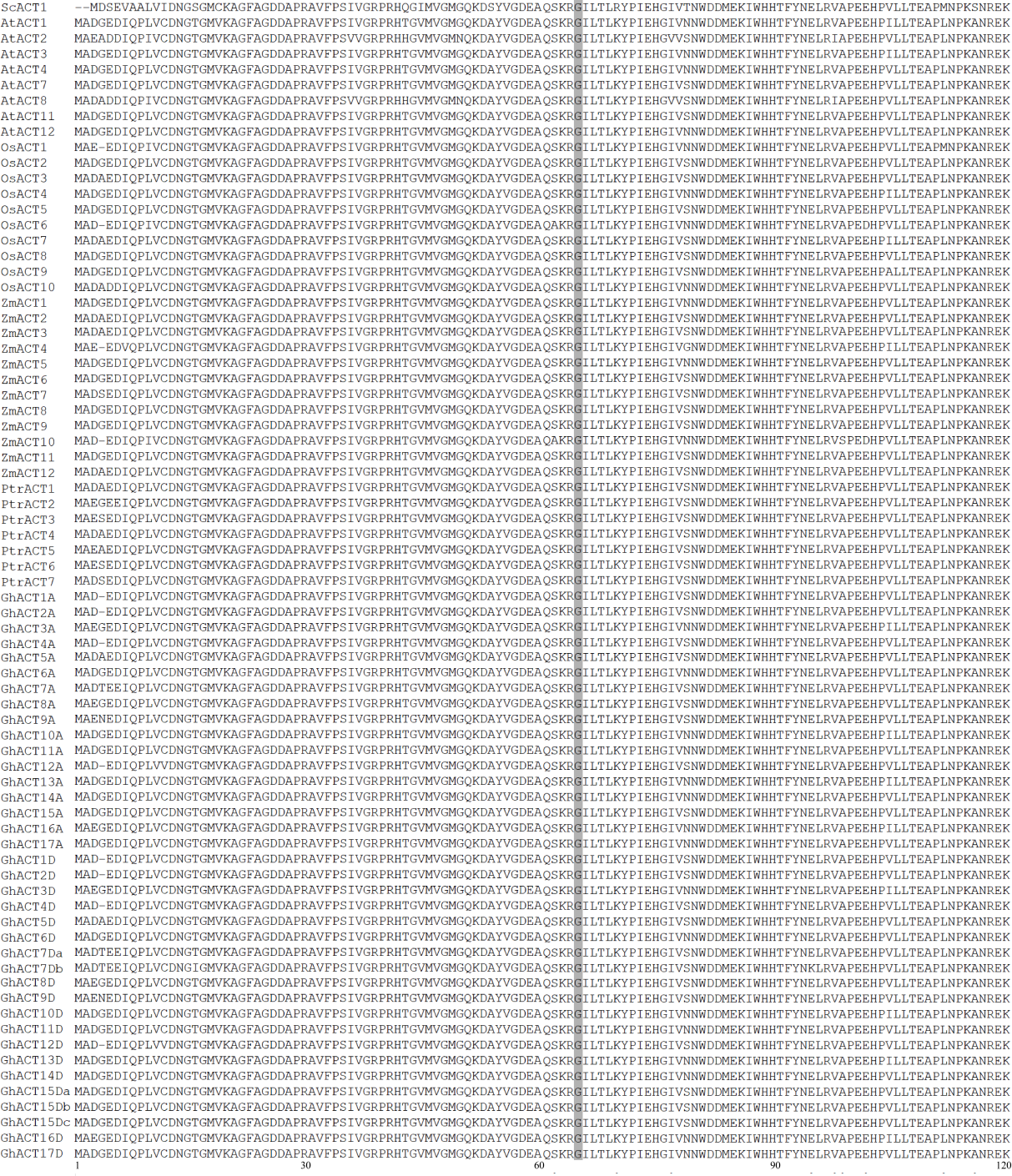


**FIGURE S10** Multiple alignment of 75 actin protein sequences from six organisms. The multiple alignment of 75 actin protein sequences (the 1-120 N terminal amino acids were presented) from yeast, Arabidopsis, rice, maize, poplar and upland cotton was performed by Clustal X2.0 software. The accession number of these proteins are listed in Supplementary Tables S4 and S5. The 65th amino acid from most actins and the structurally identical 63nd and 64th amino acid residues from several others are shaded.

**Supplementary Table 1 Segregation for presence and absence of the Li_1_ phenotype in five mapping populations**

| Year | Mapping populations | Total | No. W^a^ | No. L^b^ | Expected ratio | x^2^ test |
| --- | --- | --- | --- | --- | --- | --- |
| 2011 | (Hai7124×Li_1_)BC_1_ | 1858 | 942 | 916 | 1:1 | 0.34 |
| 2012 | (Hai7124×Li_1_)BC_1_ | 827 | 422 | 405 | 1:1 | 0.31 |
| 2013 | (Hai7124×Li_1_)BC_1_ | 1194 | 606 | 588 | 1:1 | 0.24 |
| Total | (Hai7124×Li_1_)BC_1_ | 3879 | 1970 | 1909 | 1:1 | 0.93 |
| 2015 | (Hai7124×Li_1_) F_2_ | 492 | 145 | 347 | 1:3 | 5.01 |
| 2016 | (Hai7124×Li_1_) F_2_ | 2674 | 790 | 1884 | 1:3 | 29.20 |
| Total | (Hai7124×Li_1_) F_2_ | 3166 | 935 | 2231 | 1:3 | 34.45 |

a: number of wild type (W) plants; b: number of Li_1_ mutant type (L) plants. x^2^ test for goodness-of-fit at 0.05 significance level (x^2^_0.05, 1_=3.84). Segregation of *Li_1_* in BC_1_ populations completely fit single dominant gene model. By contrast, the segregation of *Li_1_* in F_2_ populations deviating significantly from a single dominant-gene model, presumably due to the failure of many homozygous mutants to survive, which is in line with previously reports (Kohel et al., 1972; Rong et al., 2005; Liu et al., 2010; Jiang et al., 2015; Thyssen et al., 2015).

**REFERENCES**

Jiang, Y.R., Ding, M.Q., Cao, Y.F., Yang, F., Zhang, H., He, S.E., Dai, H.Q., Hao, H.F. and Rong, J.K. (2015). Genetic fine mapping and candidate gene analysis of the *Gossypium hirsutum* Ligon lintless-1 (*Li_1_*) mutant on chromosome 22(D). *Mol. Genet. Genomics,* 290, 2199–2211. doi: 10.1007/s00438-015-1070-2

Kohel, R.J. (1972). Linkage tests in upland cotton, *Gossypium hirsutum* L. II. *Crop Sci.* 5, 66–69. doi: 10.2135/cropsci1965.0011183X000500060029x

Liu, F.J., Liang, W.H. and Zhang, T.Z. (2010). Genetic analysis of homozygous-dominant-surviving Ligon lintless recombinants in cotton (*Gossypium hirsutum* L.). *Acta Agronomica Sinica,* 36, 2020–2027. doi: 10.1016/s1875-2780(09)60086-9

Rong, J., Pierce, G.J., Waghmare, V.N., et al. (2005). Genetic mapping and comparative analysis of seven mutants related to seed fber development in cotton. *Theor. Appl. Genet.* 111, 1137–1146. doi: 10.1007/s00122-005-0041-0

Thyssen, G.N., Fang, D.D., Turley, R.B., Florane, C., Li, P. and Naoumkina, M. (2015). Mapping-by-sequencing of Ligon-lintless-1 (Li_1_) reveals a cluster of neighboring genes with correlated expression in developing fibers of Upland cotton (*Gossypium hirsutum* L.). *Theor. Appl. Genet.* 128, 1703–1712. doi: 10.1007/s00122-015-2539-4

**Supplementary Table 2 List of primers used in this research**

| **Purpose** | **Primer names** | **Sequence (5'-3')** |
| --- | --- | --- |
| Mapping | W4571F | TCACCGGAATAGTGAAATCTG |
| Mapping | W4571R | CTACCCCAACCAAAGTGATTT |
| Mapping | W4806F | CCATGGAATTTCTTCGGAAC |
| Mapping | W4806R | TGAAGATGACGAATGTCCCA |
| Mapping | L385F | CCGAAAATACGGTTTCAGCACT |
| Mapping | L385R | AGCTCTTTTGGCTTGATTTTTCTGA |
| Mapping | L388F | GATTGGACACCTTCCCAGCA |
| Mapping | L388R | CCCGATTCGTTCCTCACACA |
| Mapping | L397F | CCTCTCTTTTCTTGCAGCCC |
| Mapping | L397R | TGTGAAATGCCTTAGTCCCCT |
| Mapping | L408F | CCACCAGATTTCAACTCACACG |
| Mapping | L408R | TGGTTGTGGCAATCGATGGA |
| Mapping | L411F | ACTCTTTACATAATGTCCCGTAAGA |
| Mapping | L411R | TCATGGGTATGCGGTTACGG |
| Mapping | L428F | GACTTGTGCCAAGCTTATGGT |
| Mapping | L428R | ACCTTGGCCAAAACATTCAAA |
| Mapping | L440F | CAGCAAGTGATGTAGCCGGA |
| Mapping | L440R | TCATGCTGGCATCGATGACA |
| Mapping | L447F | ATCTTCGCGAACCTCCACTG |
| Mapping | L447R | AAGGAGAAAGATGCGCACCA |
| Mapping | L451F | ACTTCAGCTGCATCCAACCA |
| Mapping | L451R | ATGACTTAAAAGAGGATTGGGT |
| Mapping | L466F | TGTCTGTGCCCGTGTAACTC |
| Mapping | L466R | GTCTACACGGCCTGAGACAC |
| Mapping | L471F | CACTACGGAACCAGCCAGTT |
| Mapping | L471R | CCCAATCCCCTTAAGGACCC |
| Mapping | L483F | GCCTCTTTGCCTTGCCAAAT |
| Mapping | L483R | GAGCAGATGAAGGCTACCTCC |
| Mapping | SNPF | GAGATGAAGCTCAATCGAAAAGTGT |
| Mapping | SNPR | ATGAAGCTCAATCGAAAAGAGG |
| Gene cloning | *Gh_D04G0859c*F | ATGGAGTTAGCCTTGAGCTTG |
| Gene cloning | *Gh_D04G0859c*R | TCATGTGTTTGAAATAGAGGAG |
| Gene cloning | *Gh_D04G0860c*F | ATGAGGATCAGGAAAAGACAG |
| Gene cloning | *Gh_D04G0860c*R | CTAGTTATTATCATCTGCTAC |
| Gene cloning | *Gh_D04G0861c*F | ATGCCAAGGAAAACTCGACATC |
| Gene cloning | *Gh_D04G0861c*R | TCAACTTCTTCCACCACTGAAAG |
| Gene cloning | *Gh_D04G0862c*F | ATGGATTCCGTCAGATCTGGTC |
| Gene cloning | *Gh_D04G0862c*R | TCATCTAGAACAGACTTTGG |
| Gene cloning | *Gh_D04G0863c*F | ATGGCAGCAAAAAGGAAGAC |
| Gene cloning | *Gh_D04G0863c*R | CTAGAAAACAAGCTCATGAG |
| Gene cloning | *Gh_D04G0864c*F | ATGGCAGCAAAAAGGAAGAC |
| Gene cloning | *Gh_D04G0864c*R | CTAACAGATTACGAAATATAAAAG |
| Gene cloning | *Gh_D04G0865c*F | ATGGCAGAAAACGAAGACATTC |
| Gene cloning | *Gh_D04G0865c*R | CTAGAAGCATTTCCTGTGCAC |
| Gene cloning | *Gh_D04G0866c*F | ATGTTTTCATGGTTGGCAAGG |
| Gene cloning | *Gh_D04G0866c*R | TTAAAGAATGTTGAAGTTTGATG |
| Gene cloning | *Gh_D04G0867c*F | ATGGGGAGTTGGAGGCGCAGC |
| Gene cloning | *Gh_D04G0867c*R | TCAAAGACAGACTACCTCCTC |
| Gene cloning | *Gh_D04G0868c*F | ATGCTTGAAGAGTTGGACATAAG |
| Gene cloning | *Gh_D04G0868c*R | TTACATGTTATCGTCCGTATTTG |
| Gene cloning | *Gh_D04G0869c*F | ATGGAACCTTTGGACCCTCAC |
| Gene cloning | *Gh_D04G0869c*R | CTATCGACATGGTGGGCGAAC |
| Genome cloning | 5'Flanking2Kb(*Ghli1*)F | ATTAAGGGTGAGCATTCAATC |
| Genome cloning | 5'Flanking2Kb(*Ghli1*)R | TTTCTATTGCCTGTTTCATAG |
| Genome cloning | 3'Flanking2Kb(*Ghli1*)F | AGAAGAGCTATACATACAGT |
| Genome cloning | 3'Flanking2Kb(*Ghli1*)R | CCTTGATTTTAACACTTGTAAT |
| qRT-PCR | qRT-*Gh_D04G0859*F | TCAGAATAGAAGAGCAAGGAC |
| qRT-PCR | qRT-*Gh_D04G0859*R | GTGGTAGCGGGTAACTGCATG |
| qRT-PCR | qRT-*Gh_D04G0860*F | CGAAGCCGATCTTTAGCATC |
| qRT-PCR | qRT-*Gh_D04G0860*R | CTCTCTTCTTTGTCATCATC |
| qRT-PCR | qRT-*Gh_D04G0861*F | GGAACTATTTATGTTAAGAAG |
| qRT-PCR | qRT-*Gh_D04G0861*R | TTCCACCAGGATGAAATCTC |
| qRT-PCR | qRT-*Gh_D04G0862*F | TTGCTTGCAAGACATCTTCC |
| qRT-PCR | qRT-*Gh_D04G0862*R | TCATCTTATGCCACATATTAG |
| qRT-PCR | qRT-*Gh_D04G0863*/*Gh_D04G0864*F | GGTGTTCCTTTACGGCGTGTG |
| qRT-PCR | qRT-Gh_D04G0863/Gh_D04G0864R | CTTGCAGCAAGGTATGCCTTC |
| qRT-PCR | qRT-*Gh_D04G0865*F | AGGAGGCTCCATTTTGGCATC |
| qRT-PCR | qRT-*Gh_D04G0865*R | AAGCAACACTGGAAACCAAC |
| qRT-PCR | qRT-*Gh_D04G0866*F | ACAACCTAGTGATAAGTTTC |
| qRT-PCR | qRT-*Gh_D04G0866*R | GACAACAACCGTAATATCATC |
| qRT-PCR | qRT-*Gh_D04G0867*F | TATAGCTCGCAGAAACAAGC |
| qRT-PCR | qRT-*Gh_D04G0867*R | AACCTTCCTTTGATCCATGC |
| qRT-PCR | qRT-*Gh_D04G0868*F | GAAGTTCCACCAAGAGAAGC |
| qRT-PCR | qRT-*Gh_D04G0868*R | GAACGGCCAACAAATCGAGC |
| qRT-PCR | qRT-*Gh_D04G0869*F | GCTGACCCTATCAAAGGAAC |
| qRT-PCR | qRT-*Gh_D04G0869*R | GGAACGCGCATGGATACACTG |
| qRT-PCR | qRT-*GhACT3D*F | ATTGGTGGCTCCATATTAGC |
| qRT-PCR | qRT-*GhACT3D*R | TTGCAAGAAACCTTAGACAG |
| qRT-PCR | qRT-*GhACT8A*F | AGTACAGTGTCTGGATCGGAG |
| qRT-PCR | qRT-*GhACT8A*R | TCATTGTTTCACTCCAACATG |
| qRT-PCR | qRT-*GhACT11A*F | ATTGGAGGCTCCATCTTAGC |
| qRT-PCR | qRT-*GhACT11A*R | GAAGCTTGCGAGCTGCAATTG |
| qRT-PCR | qRT-*GhACT14A*F | TCTGGATCGGTGGCTCGATC |
| qRT-PCR | qRT-*GhACT14A*R | AGAACAAAGCAGAGCTGCAC |
| qRT-PCR | qRT-*GhACT15A*F | ATCTATCTTGGCATCACTCAG |
| qRT-PCR | qRT-*GhACT15A*R | AAGTACACCATCCTACAATG |
| qRT-PCR | qRT-*GhACT5D*F | GTACAGTGTCTGGATTGGAG |
| qRT-PCR | qRT-*GhACT5D*R | CAATAATCCATTTCCCACATC |
| qRT-PCR | qRT-*GhACT1D*F | CTTGCACCTAGCAGCATGAAG |
| qRT-PCR | qRT-*GhACT1D*R | AGGCAACCGAACTGAACTCG |
| qRT-PCR | qRT-*GhACT12A*F | ATACAGTGTCTGGATTGGTG |
| qRT-PCR | qRT-*GhACT12A*R | CCATATCTCATACATTCATG |
| qRT-PCR | qRT-*GhACT2A*F | TAGCAGCATGAAGATCAAGG |
| qRT-PCR | qRT-*GhACT2A*R | ATCTAATGAGAGACAGACTG |
| qRT-PCR | qRT-*GhACT4D*F | AGTGTCTGGATCGGAGGATC |
| qRT-PCR | qRT-*GhACT4D*R | ATGCAAAATACAGTACAAGC |
| qRT-PCR | qRT-*GhACT7A*F | GTCTGGATTGGAGGATCTATC |
| qRT-PCR | qRT-*GhACT7A*R | GTACATCCTCCTTCAGTGATC |
| qRT-PCR | qRT-*GhACT16D*F | AGTATAGTGTCTGGATTGGC |
| qRT-PCR | qRT-*GhACT16D*R | ATGTTTGATCGTTCTATCCG |
| qRT-PCR | qRT-*His3*F | CGGTGGTGTGAAGAAGCCTCAT |
| qRT-PCR | qRT-*His3*R | AATTTCACGAACAAGCCTCTGGAA |
| VIGS | pTRV2::CDSF | CTACCTGATGGACAGGTTATTA |
| VIGS | pTRV2::CDSR | CTGTGCACAATTGATGGCCCAG |
| VIGS | pTRV2::UTRF | AGAAGAGCTATACATACAG |
| VIGS | pTRV2::UTRR | CAAGAGAAAATAGTTCTTC |
| Prokaryotic expression | pET30A::*GhLi1*/*Ghli1*F | GCAGAAAACGAAGACATTC |
| Prokaryotic expression | pET30A::*GhLi1*/*Ghli1*R | GAAGCATTTCCTGTGCAC |

**Supplementary Table 3 List of eleven annotated genes in the mapping region**

| **ID** | **Functional description** |
| --- | --- |
| *Gh_D04G0859* | Homeobox-leucine zipper protein HAT14 |
| *Gh_D04G0860* | Growth-regulating factor 10 |
| *Gh_D04G0861* | Cysteine/Histidine-rich C1 domain family protein |
| *Gh_D04G0862* | Tubulin beta-7 chain |
| *Gh_D04G0863* | Ribosomal protein L6 family protein |
| *Gh_D04G0864* | Ribosomal protein L6 family protein |
| *Gh_D04G0865* | Actin 1 |
| *Gh_D04G0866* | Protein phosphatase 2C family protein |
| *Gh_D04G0867* | Nucleotidylyl transferase superfamily protein |
| *Gh_D04G0868* | Plant intracellular ras group-related LRR 4 |
| *Gh_D04G0869* | Peptidase M1 family protein |

**Supplementary Table 4 Actin family genes in *G. hirsutum***

| **Name** | **ID** | **Protein length** | **Name** | **ID** | **Protein length** |
| --- | --- | --- | --- | --- | --- |
| *GhACT1A* | *Gh_A01G0009* | 376 | *GhACT1D* | *Gh_D01G0008* | 376 |
| *GhACT2A* | *Gh_A01G1475* | 376 | *GhACT2D* | *Gh_D01G1711* | 376 |
| *GhACT3A* | *Gh_A01G1915* | 377 | *GhACT3D* | *Gh_D01G2174* | 377 |
| *GhACT4A* | *Gh_A03G1858* | 376 | *GhACT4D* | *Gh_D02G2297* | 376 |
| *GhACT5A* | *Gh_A11G3240* | 377 | *GhACT5D* | *Gh_D11G3453* | 377 |
| *GhACT6A* | *Gh_A03G2078* | 377 | *GhACT6D* | *Gh_D03G1211* | 377 |
| *GhACT7A* | *Gh_A05G3359* | 377 | *GhACT7Da* | *Gh_D04G0254* | 377 |
|  |  |  | *GhACT7Db* | *Gh_D04G0257* | 377 |
| *GhACT8A* | *Gh_A05G3047* | 377 | *GhACT8D* | *Gh_D04G0598* | 377 |
| *GhACT9A* | *Gh_A04G1367* | 377 | *GhACT9D* | *Gh_D04G0865* | 377 |
| *GhACT10A* | *Gh_A07G0895* | 377 | *GhACT10D* | *Gh_D07G0965* | 377 |
| *GhACT11A* | *Gh_A08G0002* | 377 | *GhACT11D* | *Gh_D08G0042* | 377 |
| *GhACT12A* | *Gh_A08G0945* | 376 | *GhACT12D* | *Gh_D08G1154* | 376 |
| *GhACT13A* | *Gh_A09G1461* | 377 | *GhACT13D* | *Gh_D09G1470* | 377 |
| *GhACT14A* | *Gh_A10G1961* | 377 | *GhACT14D* | *Gh_D10G2243* | 377 |
| *GhACT15A* | *Gh_Sca008940G01* | 377 | *GhACT15Da* | *Gh_D11G0591* | 377 |
|  |  |  | *GhACT15Db* | *Gh_D11G0592* | 377 |
|  |  |  | *GhACT15Dc* | *Gh_D11G0593* | 377 |
| *GhACT16A* | *Gh_A13G0187* | 377 | *GhACT16D* | *Gh_D13G0202* | 377 |
| *GhACT17A* | *Gh_A13G1982* | 377 | *GhACT17D* | *Gh_D13G2381* | 377 |

**Supplementary Table 5 Actins used for multiple alignment**

| **ID** | **Name** | **ID** | **Name** |
| --- | --- | --- | --- |
| Os01g64630 | OsACT1 | Potri.006G192700 | PtrACT3 |
| Os01g73310 | OsACT2 | Potri.008G055500 | PtrACT4 |
| Os03g50885 | OsACT3 | Potri.010G204300 | PtrACT5 |
| Os03g61970 | OsACT4 | Potri.016G045500 | PtrACT6 |
| Os05g01600 | OsACT5 | Potri.018G128300 | PtrACT7 |
| Os05g36290 | OsACT6 | Potri.019G010400 | PtrACT8 |
| Os10g36650 | OsACT7 | GRMZM2G006765 | ZmACT1 |
| Os11g06390 | OsACT8 | GRMZM2G030169 | ZmACT2 |
| Os12g06660 | OsACT9 | GRMZM2G047055 | ZmACT3 |
| Os12g44350 | OsACT10 | GRMZM2G053284 | ZmACT4 |
| AT2G37620 | AtACT1 | GRMZM2G053299 | ZmACT5 |
| AT3G18780 | AtACT2 | GRMZM2G067985 | ZmACT6 |
| AT3G53750 | AtACT3 | GRMZM2G082484 | ZmACT7 |
| AT5G59370 | AtACT4 | GRMZM2G104017 | ZmACT8 |
| AT5G09810 | AtACT7 | GRMZM2G110378 | ZmACT9 |
| AT1G49240 | AtACT8 | GRMZM2G126010 | ZmACT10 |
| AT3G12110 | AtACT11 | GRMZM2G126190 | ZmACT11 |
| AT3G46520 | AtACT12 | GRMZM2G152328 | ZmACT12 |
| Potri.001G309500 | PtrACT1 | AAD38853 | ScACT1 |
| Potri.001G453600 | PtrACT2 |  |  |

**Supplementary Table 6** Detailed data of the time course of actin polymerization monitored by pyrene fluorescence

| Time (second) | actin alone | actin + 0.1 µM Ghli_1_ | actin + 0.2 µM Ghli_1_ | actin + 0.1 µM GhLi_1_ | actin + 0.2 µM GhLi_1_ |
| --- | --- | --- | --- | --- | --- |
| 0 | 1.494 ± 0.0035 | 1.490 ± 0.0026 | 1.491 ± 0.0053 | 1.492 ± 0.0026 | 1.493 ± 0.0026 |
| 50 | 1.498 ± 0.0017 | 1.562 ± 0.0036 | 1.576 ± 0.0087^*^ | 1.495 ± 0.0035 | 1.496 ± 0.0017 |
| 100 | 1.634 ± 0.0235 | 1.652 ± 0.0075 | 1.715 ± 0.0089^*^ | 1.574 ± 0.0044^*^ | 1.499 ± 0.0030^**^ |
| 150 | 1.737 ± 0.0132 | 1.749 ± 0.0144 | 1.822 ± 0.0147^*^ | 1.665 ± 0.0350^*^ | 1.503 ± 0.0072^**^ |
| 200 | 1.847 ± 0.0212 | 1.855 ± 0.0131 | 1.955 ± 0.0275^*^ | 1.752 ± 0.0056^*^ | 1.562 ± 0.004^**^ |
| 250 | 1.973 ± 0.0106 | 2.027 ± 0.0144^*^ | 2.133 ± 0.0193^**^ | 1.836 ± 0.0087^**^ | 1.595 ± 0.0062^**^ |
| 300 | 2.108 ± 0.0149 | 2.201 ± 0.0118^*^ | 2.322 ± 0.0265^**^ | 1.912 ± 0.0087^**^ | 1.628 ± 0.0044^**^ |
| 350 | 2.233 ± 0.0695 | 2.341 ± 0.0140^*^ | 2.519 ± 0.0226^**^ | 1.996 ± 0.0330^**^ | 1.645 ± 0.0046^**^ |
| 400 | 2.377 ± 0.0344 | 2.511 ± 0.0225^**^ | 2.721 ± 0.0261^**^ | 2.075 ± 0.0178^**^ | 1.677 ± 0.0044^**^ |
| 450 | 2.541 ± 0.0185 | 2.721 ± 0.0217^**^ | 2.968 ± 0.0590^**^ | 2.171 ± 0.0347^**^ | 1.716 ± 0.0050^**^ |
| 500 | 2.715 ± 0.0148 | 2.937 ± 0.0236^**^ | 3.237 ± 0.0949^**^ | 2.262 ± 0.0233^**^ | 1.763 ± 0.0040^**^ |
| 550 | 2.901 ± 0.0364 | 3.154 ± 0.0437^**^ | 3.481 ± 0.0529^**^ | 2.342 ± 0.0131^**^ | 1.792 ± 0.0036^**^ |
| 600 | 3.118 ± 0.0123 | 3.404 ± 0.0181^**^ | 3.757 ± 0.0478^**^ | 2.437 ± 0.0166^**^ | 1.834 ± 0.0079^**^ |
| 650 | 3.299 ± 0.0488 | 3.616 ± 0.0173^**^ | 4.011 ± 0.0118^**^ | 2.539 ± 0.0374^**^ | 1.85 ± 0.0171^**^ |
| 700 | 3.497 ± 0.0350 | 3.866 ± 0.0409^**^ | 4.325 ± 0.0265^**^ | 2.652 ± 0.0095^**^ | 1.871 ± 0.0053^**^ |
| 750 | 3.722 ± 0.0199 | 4.113 ± 0.1035^**^ | 4.611 ± 0.0125^**^ | 2.761 ± 0.0349^**^ | 1.923 ± 0.0092^**^ |
| 800 | 3.959 ± 0.0353 | 4.377 ± 0.0817^**^ | 4.916 ± 0.0156^**^ | 2.874 ± 0.0166^**^ | 1.977 ± 0.0040^**^ |
| 850 | 4.221 ± 0.0846 | 4.621 ± 0.1244^**^ | 5.273 ± 0.1229^**^ | 2.995 ± 0.0151^**^ | 1.993 ± 0.0072^**^ |
| 900 | 4.484 ± 0.0348 | 4.923 ± 0.0208^**^ | 5.662 ± 0.1808^**^ | 3.101 ± 0.0087^**^ | 2.021 ± 0.0046^**^ |
| 950 | 4.754 ± 0.0511 | 5.202 ± 0.0501^**^ | 6.025 ± 0.0754^**^ | 3.233 ± 0.0203^**^ | 2.076 ± 0.0165^**^ |
| 1000 | 5.021 ± 0.1143 | 5.561 ± 0.0556^**^ | 6.438 ± 0.1398^**^ | 3.368 ± 0.0207^**^ | 2.121 ± 0.0061^**^ |
| 1050 | 5.282 ± 0.1492 | 5.858 ± 0.0512^**^ | 6.831 ± 0.0366^**^ | 3.515 ± 0.0560^**^ | 2.145 ± 0.0056^**^ |
| 1100 | 5.562 ± 0.1756 | 6.188 ± 0.1403^**^ | 7.252 ± 0.1216^**^ | 3.654 ± 0.0281^**^ | 2.190 ± 0.0082^**^ |
| 1150 | 5.871 ± 0.1698 | 6.478 ± 0.1038^**^ | 7.680 ± 0.2733^**^ | 3.824 ± 0.0790^**^ | 2.244 ± 0.0046^**^ |
| 1200 | 6.173 ± 0.1589 | 6.836 ± 0.1766^**^ | 8.122 ± 0.2245^**^ | 4.023 ± 0.1214^**^ | 2.330 ± 0.0149^**^ |

Data represent the means ± SD from three biological replicates. Student’s *t*-test: **P* < 0.05, ***P* < 0.01.
